# Cholesterol‐27α‐hydroxylase inhibitor nilvadipine can effectively treat cholestatic liver injury in adult offspring induced by prenatal dexamethasone exposure

**DOI:** 10.1002/mco2.70110

**Published:** 2025-03-04

**Authors:** Wen Hu, Jiayong Zhu, Qi Zhang, Xiaoqian Lu, Luting Yu, Bin Li, Liaobin Chen, Hui Wang

**Affiliations:** ^1^ Department of Pharmacology Wuhan University School of Basic Medical Sciences Wuhan China; ^2^ Department of Pharmacy Zhongnan Hospital of Wuhan University Wuhan China; ^3^ Hubei Provincial Key Laboratory of Developmentally Originated Disease Wuhan China; ^4^ Department of Orthopedic Surgery Zhongnan Hospital of Wuhan University Wuhan China

**Keywords:** cholestatic liver injury, cholesterol 27α‐hydroxylase, dexamethasone, miR‐450b‐3p, nilvadipine

## Abstract

Prenatal dexamethasone exposure (PDE) can increase offspring susceptibility to various diseases. However, the pathogenesis and early prevention for PDE offspring prone to cholestatic liver injury have been unclear. In this study, we collected human umbilical cord blood from neonates with prenatal dexamethasone therapy, showing increased primary unconjugated bile acid levels in utero. PDE increased blood primary bile acid levels, enhanced endoplasmic reticulum stress, and led to cholestatic liver injury in adulthood in rats, which is accompanied by the persistent increase of H3K14ac level in cholesterol 27α‐hydroxylase (CYP27A1) promoter and its expression before and after birth. In vitro, dexamethasone activates glucocorticoid receptors, binding to the CYP27A1 promoter, and promotes its transcriptional expression. Through the miR‐450b‐3p/SIRT1 pathway, it increased the H3K14ac level of the CYP27A1 promoter to enhance its transcription, which continues after birth. Finally, nilvadipine effectively reversed cholestatic liver injury induced by PDE. This study confirmed PDE could cause cholestatic liver injury, and innovatively proposed its early intervention target (CYP27A1) and effective drug (nilvadipine), providing a theoretical and experimental basis for guiding rational drug use during pregnancy, and preventing and treating the fetal‐originated cholestatic liver injury.

## Graphical abstract

Dexamethasone enhanced CYP27A1 promoter H3K14ac level and expression through the GR/miR‐450b‐3p/SIRT1 pathway during pregnancy. The elevated CYP27A1 persists until birth, which elevates primary unconjugated bile acids and endoplasmic reticulum stress, causing cholestatic liver injury in adult female offspring. CYP27A1 and nilvadipine are first proposed as early intervention targets and effective drugs against fetal‐originated cholestatic liver injury.

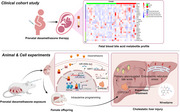

## 1 INTRODUCTION

Epidemiological investigations have shown that multiple adult diseases originate in fetal life.^1^, ^2^, ^3^ Dexamethasone, a synthetic glucocorticoid, is frequently utilized in imminent preterm labor to hasten fetal lung development and decrease the risk of neonatal respiratory distress syndrome.^4^, ^5^, ^6^ A WHO survey across 29 nations, encompassing 359 facilities, revealed that synthetic glucocorticoid prophylaxis in preterm infants (22–36 weeks) was administered at a rate of 54%, with usage peaking at 91% in select countries.^7^ Nevertheless, dexamethasone in pregnancy has both beneficial and detrimental effects, leading to low offspring birth weight, impaired multiorgan development, and increased susceptibility to chronic diseases.^8^, ^9^, ^10^ Consequently, the prenatal safety of dexamethasone is a subject of worldwide research interest.

Cholestatic liver injury (CLI), induced by abnormal bile formation or flow, is often secondary to drug‐induced liver injury.^11^ Studies have found that prenatal dexamethasone therapy (PDT) increased blood total bile acid (TBA) levels in infants.^12^, ^13^ Our research indicates that prenatal dexamethasone exposure (PDE) can alter bile acid metabolic profiles in offspring rats and cause liver injury in adulthood.^14^ PDE reduces fetal blood bile acid outflow to the mother by inhibiting placental bile acid transport, leading to increased levels of primary bile acids.^15^ As “bad” bile acids, hydrophobic bile acids are a critical cause of liver injury when they accumulate in hepatocytes.^16^, ^17^, ^18^ It has been found that the accumulation of bile acids can cause endoplasmic reticulum stress (ERS) and mitochondrial damage by enhancing glucose‐regulatory protein 78 (GRP78), thus leading to liver injury.^19^

Intrauterine programming denotes lasting alterations in fetal tissues and organs due to in utero damage, contributing to a range of diseases with fetal origins.^1^, ^2^, ^3^ The liver synthesizes bile acids from cholesterol via enzymes like cholesterol 7α‐hydroxylase (CYP7A1) and cholesterol 27α‐hydroxylase (CYP27A1).^20^ In adults, bile acid synthesis mainly involves CYP7A1, while in fetuses, it primarily involves CYP27A1.^21^ Epigenetic modifications of development‐related genes persistently impact fetal development before and after birth.^22^, ^23^ Our previous studies confirm that epigenetic modifications, such as miRNA and histone acetylation, are involved in PDE‐induced developmental programming and various adult diseases.^24^, ^25^, ^26^ Therefore, epigenetic programming of CYP27A1 might be involved in the pathogenesis of CLI in adult offspring with PDE.

This study collected umbilical cord blood from neonates with PDT and blood from PDE offspring rats to demonstrate abnormal bile acid metabolism and CLI. Using human Wharton's Jelly mesenchymal stem cell (WJ‐MSC)‐differentiated hepatoid cells and HepG2 cells, we explored the effects of dexamethasone on liver bile acid metabolizing enzymes (like CYP27A1) and related epigenetic mechanisms. Serial of experiments identified early intervention targets and effective drugs for preventing CLI in adult PDE offspring. This study provides theoretical and practical insights into clarifying intrauterine programming mechanisms behind PDE‐induced CLI, guiding rational drug use during pregnancy, and developing an early prevention strategy.

## 2 RESULTS

### 2.1 Clinical PDT increased serum primary and unconjugated bile acid levels in neonates

**FIGURE 1:**
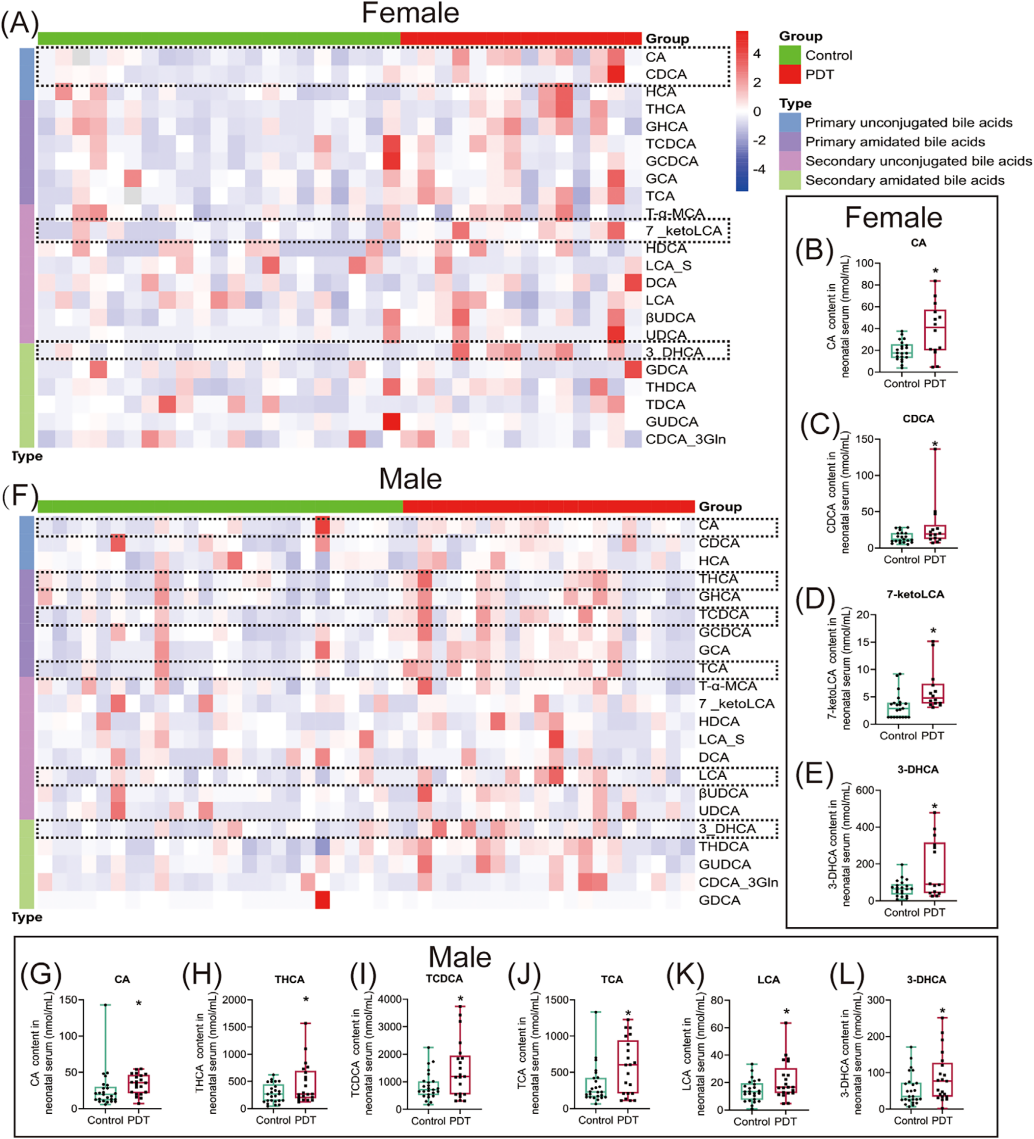
Changes in neonatal serum targeted bile acid metabolic profiles in clinical with prenatal dexamethasone therapy (PDT). (A, F) Heatmap of female or male serum bile acid metabolic profiles; (B‐E) Differential content of bile acids in female serum. (G–L) Differential content of bile acids in male serum. Data are shown as the mean ± SEM, *n* = 21 for the experiment. ^*^ *p *< 0.05, ^**^ *p *< 0.01 vs. control. CA, cholic acid; CDCA, deoxycholic acid; HCA, hyocholic acid; THCA, taurohyocholic acid; GHCA, glycohyocholic acid; TCDCA, taurochenodeoxycholic acid; GCDCA, glycochenodeoxycholic acid; GCA, glycocholic acid; TCA, taurocholic acid; T‐α‐MCA, tauro‐alpha‐muricholic acid; 7_ketoLCA, 7‐ketolithocholic acid; HDCA, hyodeoxycholic acid; LCA_S, lithocholic acid 3‐sulfate; DCA, deoxycholic acid; LCA, lithocholic acid; βUDCA, β‐ursodeoxycholic acid; UDCA, ursodeoxycholic acid; 3_DHCA, 3‐dehydrocholic acid; THDCA, taurohyodeoxycholic acid; TDCA, taurodeoxycholic acid; GUDCA, glycoursodeoxycholic acid; CDCA_3Gln, chenodeoxycholic acid‐3‐beta‐d‐glucuronide; GDCA, glycodeoxycholic acid.

We collected the neonate umbilical cord blood and conducted a targeted bile acid metabolic profile analysis using liquid chromatography‐mass spectrometry (LC‐MS) technology. The general information on the subjects is shown in Table S1. Compared with the control group, the levels of serum primary unconjugated bile acids, cholic acid (CA), and chenodeoxycholic acid (CDCA), were significantly increased in female PDT neonates (Figure 1A–C), and the levels of secondary unconjugated bile acids, 7‐ketolithocholic acid (7‐ketoLCA) and 3‐dehydrocholic acid (3‐DHCA) were elevated (Figure 1D,E). In the male PDT neonates, the levels of serum CA as well as primary bound bile acids, taurohyocholic acid (THCA), taurochenodeoxycholic acid (TCDCA), and taurocholic acid (TCA) were significantly higher than those in the control group (Figure 1F–J), and the levels of secondary bound bile acids lithocholic acid (LCA) and 3‐DHCA were also significantly increased (Figure 1K,L). In conclusion, the primary characteristic of serum bile acid metabolic changes in PDT neonates is the increase of primary unconjugated bile acid levels.

### 2.2 PDE‐induced ERS enhancement and CLI in female (rather than male) adult offspring rats

**FIGURE 2:**
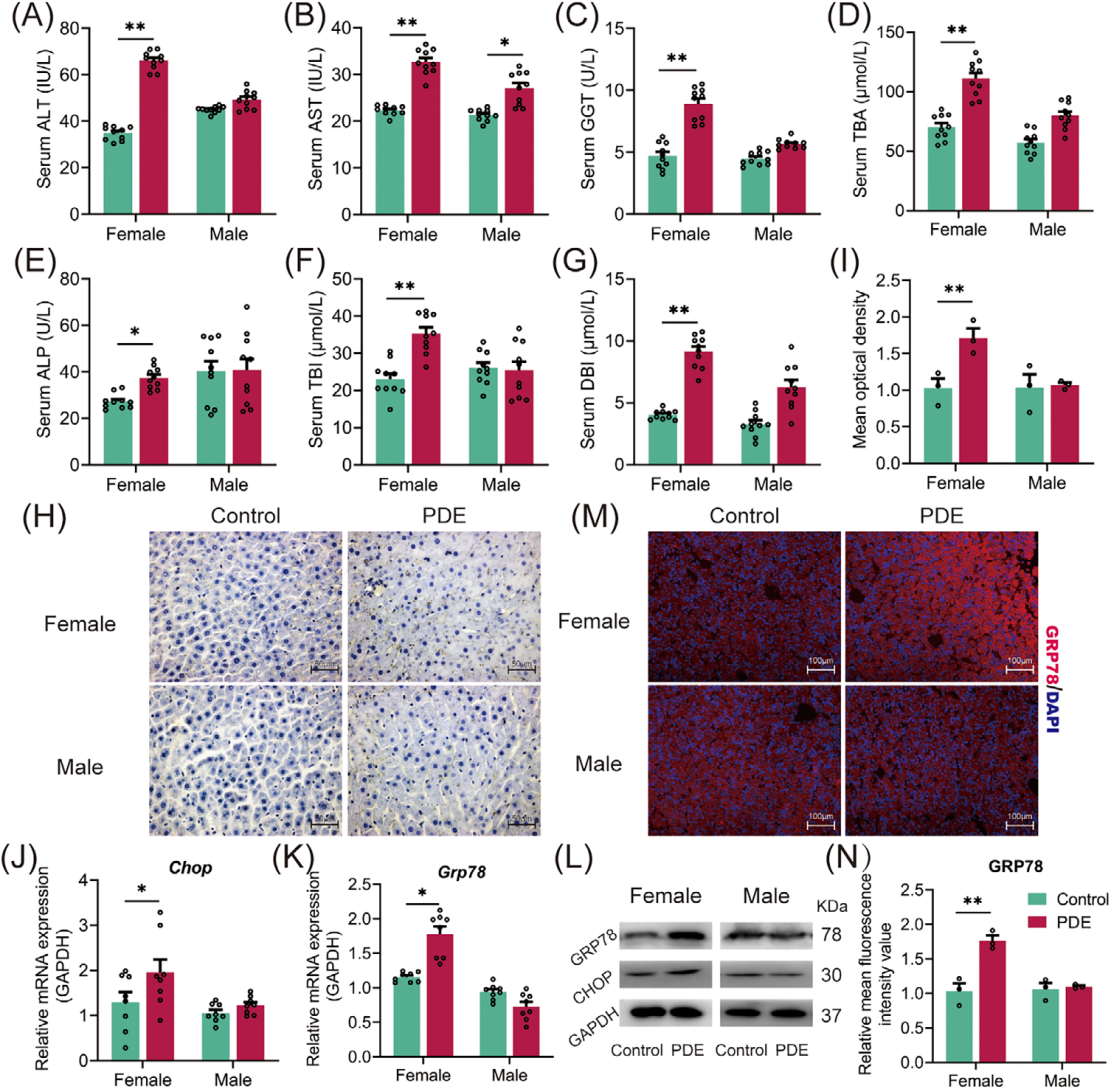
Changes in related indicators of endoplasmic reticulum stress and cholestatic liver injury in female and male adult offspring rats at postnatal week 28 with PDE. (A–G) Serum ALT, AST, GGT, TBA, ALP, TBI, and DBI levels. (H) Representative rhodanine staining images(400×); (I) Mean optical density value of rhodanine staining. (J, K) CHOP and GRP78 mRNA expression; (L) CHOP and GRP78 protein expression. (M, N) Representative immunoblots and quantification of GRP78 (200×). Data are shown as the mean ± SEM, *n* = 3 for rhodanine staining, western blot, and immunofluorescence, *n* = 6 for other experiments. ^*^ *p <* 0.05, ^**^ *p <* 0.01 vs. control. PDE, prenatal dexamethasone exposure; ALT, alanine aminotransferase; AST, aspartate aminotransferase; GGT, glutamyl transpeptidase; ALP, alkaline phosphatase; TBI, total bilirubin; DBI, direct bilirubin; TBA, total bile acid; CHOP, C/EBP homologous protein; GRP78, glucose‐regulated protein 78; GAPDH, glyceraldehyde‐3‐phosphate dehydrogenase.

To investigate the long‐term effect of PDE on the liver bile acid metabolism in offspring, we injected subcutaneously 0.2 mg/kg·d dexamethasone into pregnant rats at gestational day (GD)9‐20, and the indicators related to CLI were assessed in female and male offspring at postnatal week (PW)28. Compared with the control, liver injury indicators—serum alanine aminotransferase (ALT), aspartate aminotransferase (AST), and glutamyl transpeptidase (GGT) levels (Figure 2A–C), and cholestasis indicators—serum alkaline phosphatase (ALP), total bilirubin (TBI) and direct bilirubin (DBI) levels (Figure 2E–G) were significantly higher in the PDE female offspring rats. In the PDE male offspring, only the serum level of AST was increased (Figure 2B). PDE elevated the serum TBA level in female offspring but not males (Figure 2D). Rhodanine staining also indicated substantial liver bile accumulation in the PDE female offspring but not males (Figure 2H,I). ERS enhancement is the primary mediator of CLI. The results showed that the mRNA and protein expression of GRP78 and C/EBP homologous protein (CHOP) were increased or showed an increasing trend in the PDE females (Figure 2J–N), while there were no significant changes in GRP78 and CHOP expression in the PDE males (Figure 2J–N). These results suggest that PDE can induce ERS enhancement and CLI occurrence in female (rather than male) adult offspring rats.

### 2.3 Primary unconjugated bile acids were enriched in female offspring rats with PDE and participated in adult CLI

**FIGURE 3:**
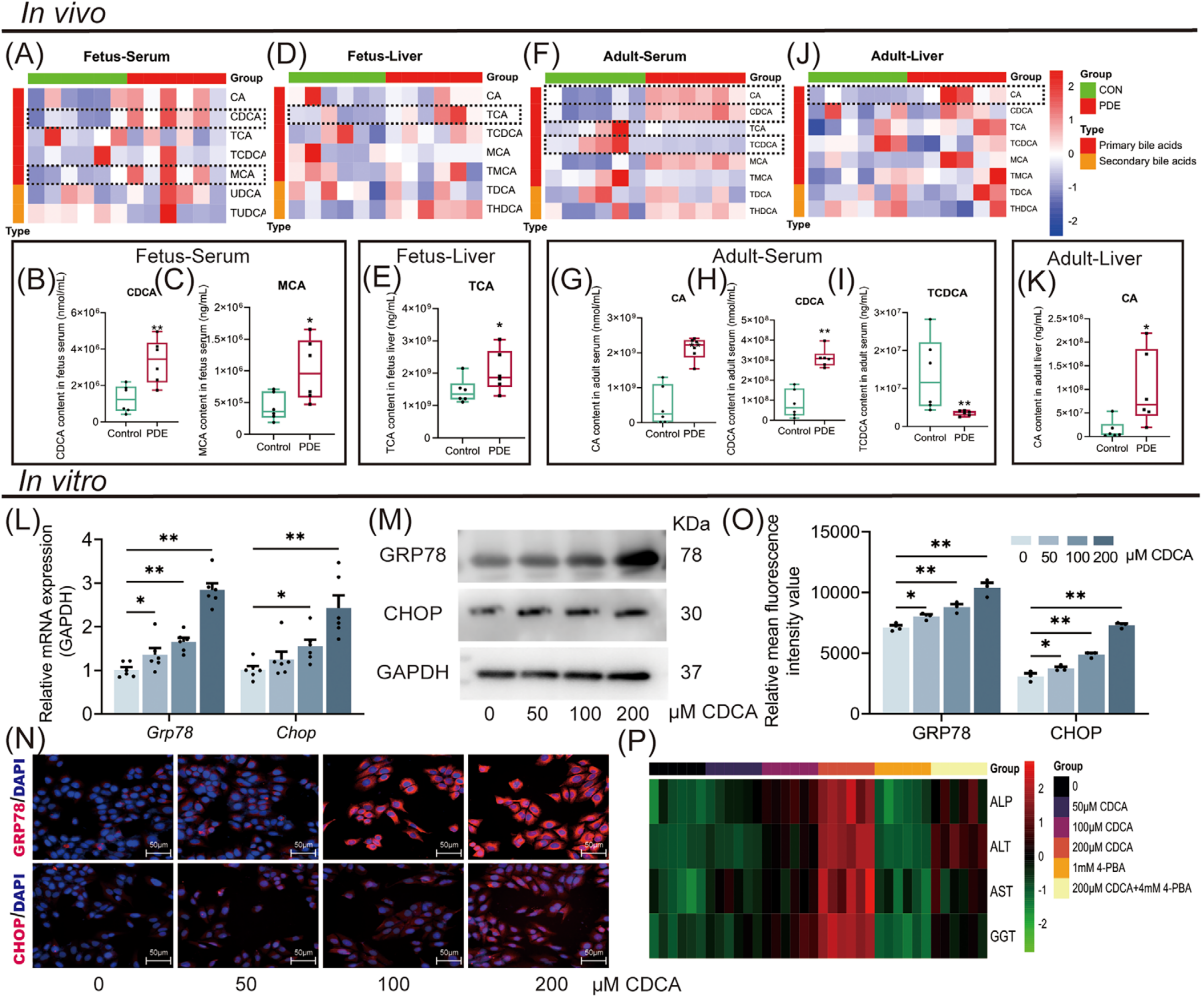
Changes in bile acid metabolic profiles in female PDE offspring rats and effects of CDCA on endoplasmic reticulum stress and cholestasis and hepatocyte injury in HepG2 cells. (A, F) Heatmap for serum bile acid metabolic profiles on GD20 and PW12. (D, J) Heatmap for liver bile acid metabolic profiles on GD20 and PW12. (B, C, G–I) Differential content of bile acids in serum on GD20 and PW12. (E, K) Differential content of bile acids in the liver on GD20 and PW12. (L) GRP78 and CHOP mRNA expression after CDCA treatment. (M) GRP78 and CHOP protein expression. (N, O) GRP78 and CHOP protein expression by immunofluorescence (400×). (P) Cholestasis and hepatocyte injury‐related indicators after 4‐PBA treatment. Data are shown as the mean ± SEM. *n* = 3 for western blot and immunofluorescence, *n* = 6 for metabolic profile, *n* = 6 for RT‐qPCR. ^*^ *p *< 0.05, ^**^ *p *< 0.01 vs. control. ^#^ *p *< 0.05, ^##^ *p *< 0.01 vs. 200 µM CDCA group. PDE, prenatal dexamethasone exposure; CDCA, deoxycholic acid; GD, gestational day; PW, postnatal week; CA, cholic acid; TCA, taurocholic acid; TCDCA, taurochenodeoxycholic acid; MCA, muricholic acid; UDCA, ursodeoxycholic acid; TUDCA, tauroursodeoxycholic acid; TMCA, tauromuricholic acid; TDCA, taurodeoxycholic acid; THDCA, tauro‐hyodeoxycholic acid; GRP78, glucose‐regulated protein 78; CHOP, C/EBP homologous protein; GAPDH, glyceraldehyde‐3‐phosphate dehydrogenase; 4‐PBA, 4‐phenylbutyrate acid; ALP, alkaline phosphatase; ALT, alanine aminotransferase; AST, aspartate aminotransferase; GGT, glutamyl transpeptidase.

To identify the type of bile acid increase in the PDE female offspring rats, we analyzed the bile acid metabolic profile changes in serum and liver tissue before and after birth using LC‐MS. On GD20, the serum CDCA, MCA levels, and liver TCA levels were higher in the PDE group compared with the control group (Figure 3A–E). At PW12, the serum CA, CDCA levels, and liver CA levels were increased while serum TCDCA level was decreased in the PDE group (Figure 3F–K). These findings indicate that PDE alters the bile acid metabolic profiles in female offspring rats both before and after birth, primarily manifested by increased levels of primary unconjugated bile acids (such as CDCA and CA).

To confirm the injurious effects of primary unconjugated bile acids on hepatocytes and elucidate the underlying mechanism, we treated HepG2 cells with different concentrations of CDCA (0, 50, 100, and 200 µM). The results showed that CDCA increased concentration‐dependently the GRP78 and CHOP expression (Figure 3L,M), while the levels of ALP, ALT, AST, and GGT in the cell supernatant were significantly elevated by CDCA (Figure 3P). Furthermore, 1mM 4‐phenylbutyrate (4‐PBA) ERS inhibitor reversed the CDCA (200 µM)‐induced cholestasis and hepatocyte injury (Figure 3P). These results suggest CDCA can promote cholestasis and hepatocyte injury by stimulating ERS.

### 2.4 PDE increased bile acid production before and after birth by upregulating liver CYP27A1 expression

**FIGURE 4:**
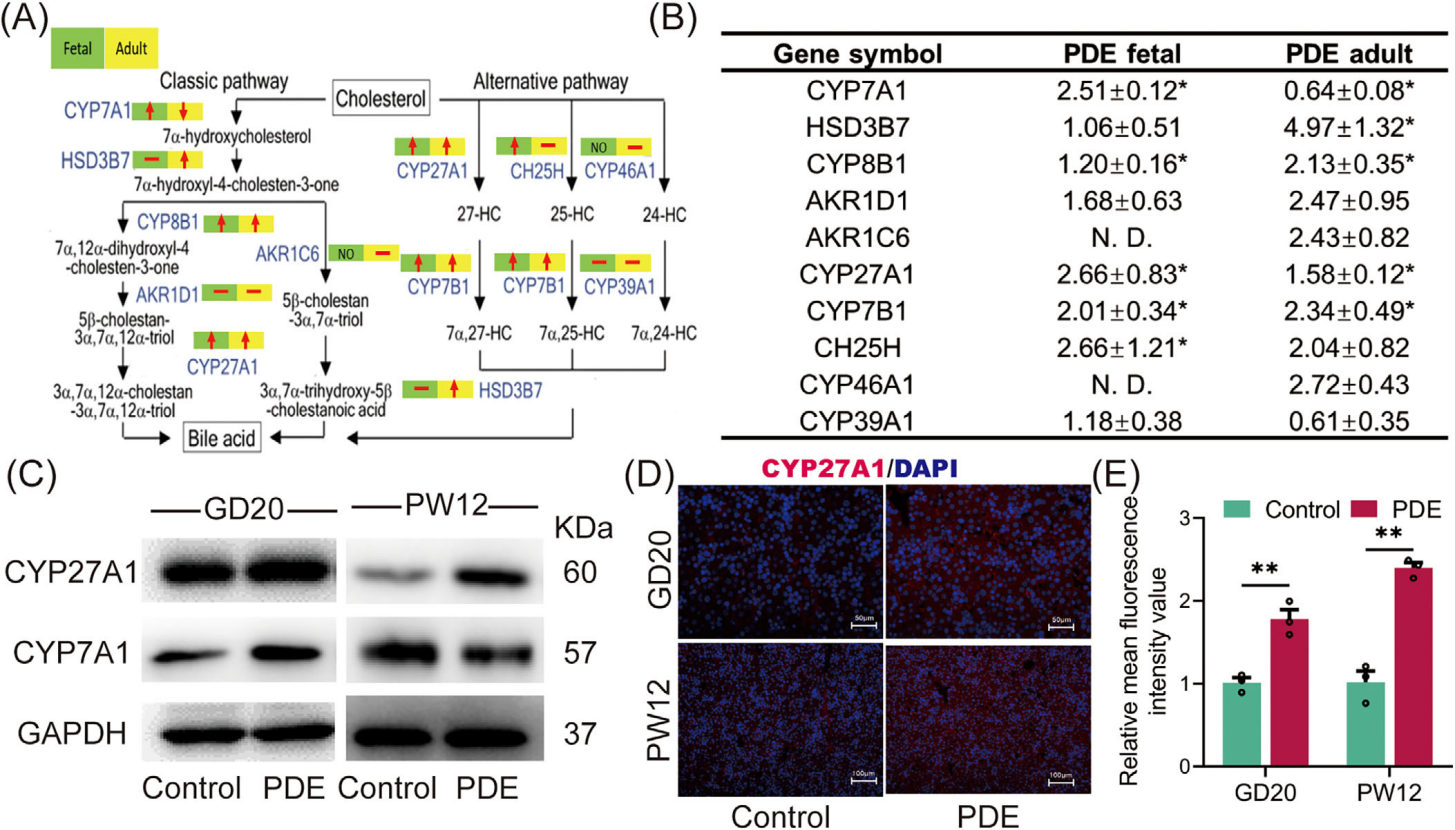
Changes in liver bile acid synthase expression in female PDE offspring rats and possible epigenetic mechanisms. (A, B) Bile acid series synthase mRNA expression on GD20 and PW12. (C) CYP27A1 and CYP7A1 protein expression on GD20 and PW12. (D, E) Representative immunoblots and quantification of CYP27A1 GD20 and PW12 (200×). Data are shown as the mean ± SEM, *n* = 3 for Western blot and immunofluorescence, *n* = 6 for RT‐qPCR. ^*^ *p <* 0.05, ^**^ *p <* 0.01 vs. control. PDE, prenatal dexamethasone exposure; GD, gestational day; PW, postnatal week; CYP7A1, cholesterol 7α‐hydroxylase; HSD3B7, hydroxy‐delta‐5‐steroid dehydrogenase, 3 beta‐ and steroid delta‐isomerase 7; CYP8B1, cytochrome P450 8B1; AKR1D1, aldo‐keto reductase family 1 member D1; AKR1C6, aldo‐keto reductase family 1, member C6; CYP27A1, cholesterol 27α‐hydroxylase; CYP7B1, cytochrome P450 7B1; CH25H, cholesterol 25‐hydroxylase; CYP46A1, cytochrome P450 family 46 subfamily A member 1; CYP39A1, cytochrome P450 39A1; GAPDH, glyceraldehyde‐3‐phosphate dehydrogenase.

We further investigated the intrauterine programming mechanism by which PDE induces elevated serum primary unconjugated bile acids in female offspring rats. The mRNA sequencing of fetal liver tissues revealed 58 genes upregulated and 19 genes down‐regulated in the PDE group compared with the control group (Figure S1A). Pathway enrichment of the differential genes identified the synthesis of primary bile acids as the most enriched pathway in the PDE group (Figure S1B). Real‐time quantitative polymerase‐chain‐reaction (RT‐qPCR) results showed significant increases in the expression of various synthases involved in both the classical and alternative bile acid synthesis pathways, including CYP27A1, CYP7A1, HSD3B7, CYP8B1, CYP7B1, and CH25H, in the PDE group at GD20 (Figure 4A,B). Among these, the protein level of CYP27A1, a key enzyme in the alternative pathway, significantly increased at GD20 and PW12 (Figure 4C), while the protein level of CYP7A1, a key enzyme in the classical pathway, was increased at GD20 but decreased at PW12 (Figure 4C). Immunofluorescence staining of liver tissues confirmed the sustained enhancement of CYP27A1 before and after birth in the PDE group (Figure 4D,E). We also examined bile acid transport and found that the changes in bile acid transport observed in utero did not persist after birth (Figure S2). Notably, we also confirmed that the CYP27A1 expression was also increased in peripheral blood mononuclear cells (PBMC) of clinical PDT neonates (Figure S3A). These findings suggest that PDE induced a sustained high expression of liver CYP27A1 and enhanced alternative bile acid synthesis pathway in the female offspring rats before and after birth.

### 2.5 Dexamethasone directly enhanced hepatocyte CYP27A1 transcription and TBA production by activating GR

**FIGURE 5:**
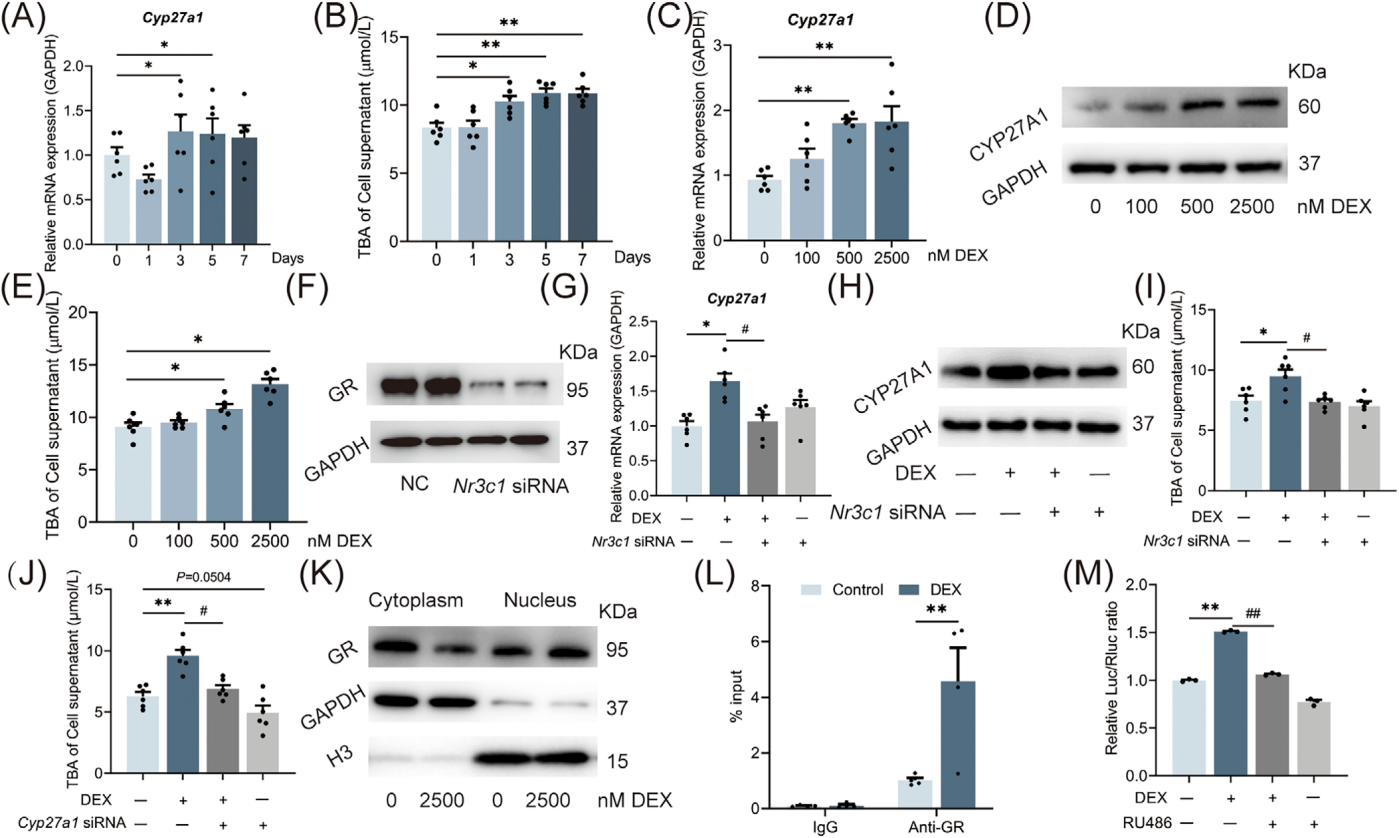
GR‐mediated CYP27A1 expression and TBA production increases in vitro induced by DEX. (A) CYP27A1 mRNA expression after DEX treatment at different times. (B) TBA level in cell supernatant after DEX treatment with different times. (C, D) CYP27A1 mRNA and protein expression after DEX treatment with different concentrations. (E) TBA levels in cell supernatant after DEX treatment with different concentrations. (F) GR protein expression after transiently transfected with 20 µM Nr3c1 siRNA or 20 µM negative control. (G, H) CYP27A1 mRNA and protein expression in the presence of Nr3c1 siRNA. (I, J) TBA level in cell supernatant in the presence of Nr3c1 siRNA and CYP27A1 siRNA. (K) GR protein expression in cytoplasm and nucleus. (L) GR enrichment in CYP27A1 promoter region. (M) The relative luciferase activities after being treated with RU486. A–F was detected in human WJ‐MSCs derived from normal newborns to differentiate into hepatocyte‐like cells. G–Q was detected in HepG2 cells. Data are shown as the mean ± SEM, *n* = 3 for western blot and ChIP assay, *n* = 6 for other experiments. ^*^ *p *< 0.05, ^**^ *p *< 0.01 vs. control; ^#^ *p *< 0.05, ^##^ *p *< 0.01 vs. 2500 nM DEX. GR, glucocorticoid receptor; CYP27A1, cholesterol 27α‐hydroxylase; TBA, total bile acid; DEX, dexamethasone; GAPDH, glyceraldehyde‐3‐phosphate dehydrogenase; H3, histone H3; WJ‐MSCs, Wharton's Jelly derived mesenchymal stem cells; ChIP, chromatin immunoprecipitation.

To confirm the effects of dexamethasone on fetal liver CYP27A1 expression and bile acid synthesis, we constructed in vitro human WJ‐MSC‐differentiated hepatoid cells. After 21 days of differentiation, the cells can not only express hepatocyte‐specific proteins (such as AFP and ALB) but also have capacities of hepatocyte glycogen synthesis (periodic acid Schiff's reaction [PAS] staining) and low‐density lipoprotein absorption (DIL‐AC‐LDL; Figure S4A). CYP27A1 mRNA expression and TBA production were increased in the cells treated with 2500 nM dexamethasone for 3 or 5 days (Figure 5A,B). We then chose an intervention time of 3 days and found that dexamethasone increased concentration‐dependently the CYP27A1 expression and TBA production (Figure 5C–E). Similarly, CYP27A1 mRNA expression and TBA production were upregulated in dexamethasone‐treated HepG2 cells (Figure S5A,B). Nr3c1 siRNA reduced the GR protein expression and reversed the increases in CYP27A1 expression and TBA production induced by dexamethasone (Figure 5F–I). Furthermore, CYP27A1 siRNA could also reverse the TBA production increase in HepG2 cells by dexamethasone (Figure 5J). These results suggested that dexamethasone can promote TBA production by binding to GR and subsequently upregulating CYP27A1 expression.

Next, we treated HepG2 cells with 2500 nM dexamethasone and found that the GR protein expression increased in the nucleus but decreased in the cytoplasm (Figure 5K). Using chromatin immunoprecipitation (ChIP)‐PCR, we confirm that dexamethasone can promote the binding of GR to the promoter region of CYP27A1 (Figure 5L). A luciferase reporter gene plasmid containing the binding site of GR as well as the CYP27A1 promoter region (between −1094 and −792) were transfected into the HepG2 cells,^27^ and the cells were treated with dexamethasone and/or RU486 (a GR antagonist) for 24 h. As expected, the transcriptional activity of the CYP27A1 promoter was significantly increased by dexamethasone but reversed by RU486 (Figure 5M). These results suggest that dexamethasone promotes the nucleus translocation of GR and its direct binding to the CYP27A1 promoter region, thus triggering the hepatocyte CYP27A1 transcription.

### 2.6 Dexamethasone increased the H3K14ac level of CYP27A1 promotor by GR/miR‐450b‐3p/SIRT1 pathway

**FIGURE 6:**
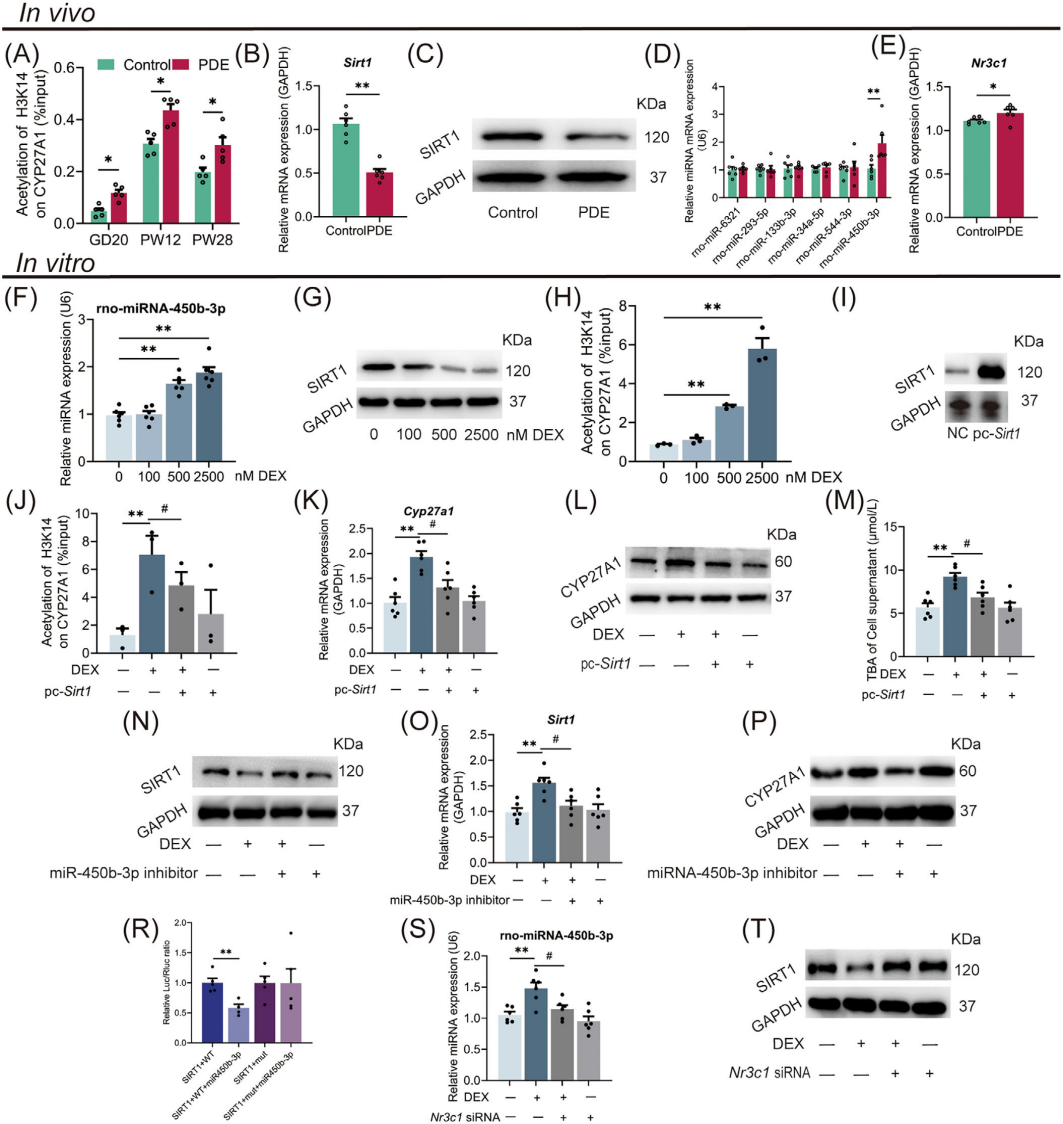
GR/miR‐450b‐3p/SIRT1 pathway mediated dexamethasone‐induced the H3K14ac level of hepatocyte CYP27A1 and its expression. (A) Enrichment of H3K14ac in CYP27A1 promoter region on GD20, PW12, and PW28. (B, C) SIRT1 mRNA and protein expression on GD20. (D) The miRNAs’ expression on GD20. (E) Nr3c1 mRNA expression on GD20. (F–H) The miR‐450b‐3p expression, SIRT1 protein expression, and H3K14ac level in CYP27A1 promoter region after DEX treatment with different concentrations. (I) SIRT1 protein expression after transiently transfected with 2.5 µg pcDNA3.1‐SIRT1 or 2.5 µg negative control. (J–M) H3K14ac level in CYP27A1 promoter region, CYP27A1 mRNA and protein expression, TBA level in cell supernatant in the presence of SIRT1 plasmid. (N–P) SIRT1 protein expression, CYP27A1 mRNA, and protein expression in the presence of miR‐450b‐3p inhibitor. (R) Target validation using luciferase reporters. The relative luciferase activities of 3′UTR reporters containing wild‐type (WT) or mutant (Mut) transcripts were assayed 24 h after co‐transfection with the indicated miRNAs or scrambled NC RNA (NC). (S, T) miR‐450b‐3p and SIRT1 protein expression in the presence of Nr3c1 siRNA. F–I was detected in the human WJ‐MSCs derived from normal newborns to differentiate into hepatocyte‐like cells. J–Y was detected in HepG2 cells. Data are shown as the mean ± SEM, *n* = 3 for Western blot and ChIP assay, *n* = 6 for other experiments. ^*^ *p <* 0.05, ^**^ *p <* 0.01 vs. control; ^#^ *p <* 0.05, ^##^ *p <* 0.01 vs. 2500 nM DEX group. GR, glucocorticoid receptor; SIRT1, Sirtuin 1; H3K14ac, histone 3 lysine 14 acetylation; CYP27A1, cholesterol 27α‐hydroxylase; GD, gestational day; PW, postnatal week; PDE, prenatal dexamethasone exposure; GAPDH, glyceraldehyde‐3‐phosphate dehydrogenase; DEX, dexamethasone; TBA, total bile acid; WJ‐MSCs, Wharton's Jelly derived mesenchymal stem cells.

To clarify how epigenetic mechanism participates in intrauterine upregulation programming of liver CYP27A1 induced by PDE, we screened the histone acetylation levels at different sites in the CYP27A1 promoter. The results showed that PDE increased the H3K14ac level in the CYP27A1 promoter before and after birth (Figure 6A), whereas its H3K9ac and H3K27ac levels were unchanged (Figure S6A,B). We further screened the histone acetylates and deacetylases in the fetal liver. The mRNA and protein expression of SIRT1, a histone deacetylase, was significantly decreased in the PDE group compared with the control (Figure 6B,C). In contrast, the expression of other acetylation‐related enzymes did not change (Figure S6C).

Glucocorticoids are known to modulate the expression of downstream target genes by regulating miRNAs. We screened the miRNAs predicted to target SIRT1 in fetal liver and found that the miR‐450b‐3p expression of PDE female fetal rats was about three times higher than the control (Figure S7A). Bioinformatics prediction showed that the 3'‐UTR of SIRT1 mRNA has a binding site for miR‐450b‐3p (Figure S7B,C). RT‐qPCR further confirmed that the miR‐450b‐3p expression in the PDE group increased on GD20 (Figure 6D) but no significant change at PW12 (Figure S7D). We also confirmed that the mRNA expression of Nr3c1 was increased in the fetal liver of the PDE group (Figure 6E). Additionally, we also identified other genes involved in bile acid synthesis and found that intrauterine changes did not last to the postnatal period (Figure S8A,B). We also detected the expression of GR/miR‐450b‐3p/SIRT1 pathway in the PDE male offspring and did not find significant changes (Figure S9A,B). In conclusion, PDE regulated the miR‐450b‐3p/SIRT1 pathway by activating GR, leading to the sustained enhancement of H3K14ac level in fetal liver CYP27A1 promoter, thereby upregulating CYP27A1 expression in the female offspring rats before and after birth.

We further verified the epigenetic mechanism of hepatocyte CYP27A1 expression upregulation induced by dexamethasone. Dexamethasone increased the miR‐450b‐3p expression, decreased SIRT1 protein expression, and increased the H3K14ac level in the CYP27A1 promoter in the WJ‐MSC‐differentiated hepatoid cells (Figure 6F–H). Increased expression of miR‐450b‐3p and decreased protein level of SIRT1 were also found in the HepG2 cells treated with dexamethasone (Figure S10A,B). To confirm that the miR‐450b‐3p/SIRT1 pathway mediates the dexamethasone‐induced H3K14ac level increase in the CYP27A1 promoter, dexamethasone‐treated HepG2 cells were further transfected with pcDNA‐SIRT1 plasmid or treated with miR‐450b‐3p inhibitor. The results showed that the SIRT1 protein expression in pcDNA‐SIRT1 transfected cells was about eight times higher than that in the control (Figure 6I), and pcDNA‐SIRT1 could reverse the increased H3K14ac level of CYP27A1 and its mRNA/protein expression, and TBA level induced by dexamethasone (Figure 6J–M). After being treated with the miR‐450b‐3p inhibitor, the decreased protein expression of SIRT1 and the increased mRNA/protein expression of CYP27A1 induced by dexamethasone were canceled (Figure 6N–P). A double luciferase reporter gene system was used to verify the influence of miR‐450b‐3p on SIRT1 expression. The HepG2 cells transfected with the wild‐type vector, but not the mutant vector of SIRT1, had a decreased firefly luciferin to renilla luciferin ratio when stimulated with miR‐450b‐3p mimics (Figure 6R), suggesting SIRT1 as a downstream target of miR‐450b‐3p. We also confirmed that Nr3c1 siRNA administration canceled the increased miR‐450b‐3p expression and decreased SIRT1 protein induced by dexamethasone (Figure 6S,T). The above results confirmed that dexamethasone promoted miR‐450b‐3p expression and its target gene SIRT1 posttranscriptional degradation by activating GR and then increased the H3K14ac level in the CYP27A1 promoter, ultimately leading to the increased CYP27A1 expression and TBA production in hepatocytes.

### 2.7 Nilvadipine reversed CLI induced by PDE and had long‐term efficacy in female offspring rats

**FIGURE 7:**
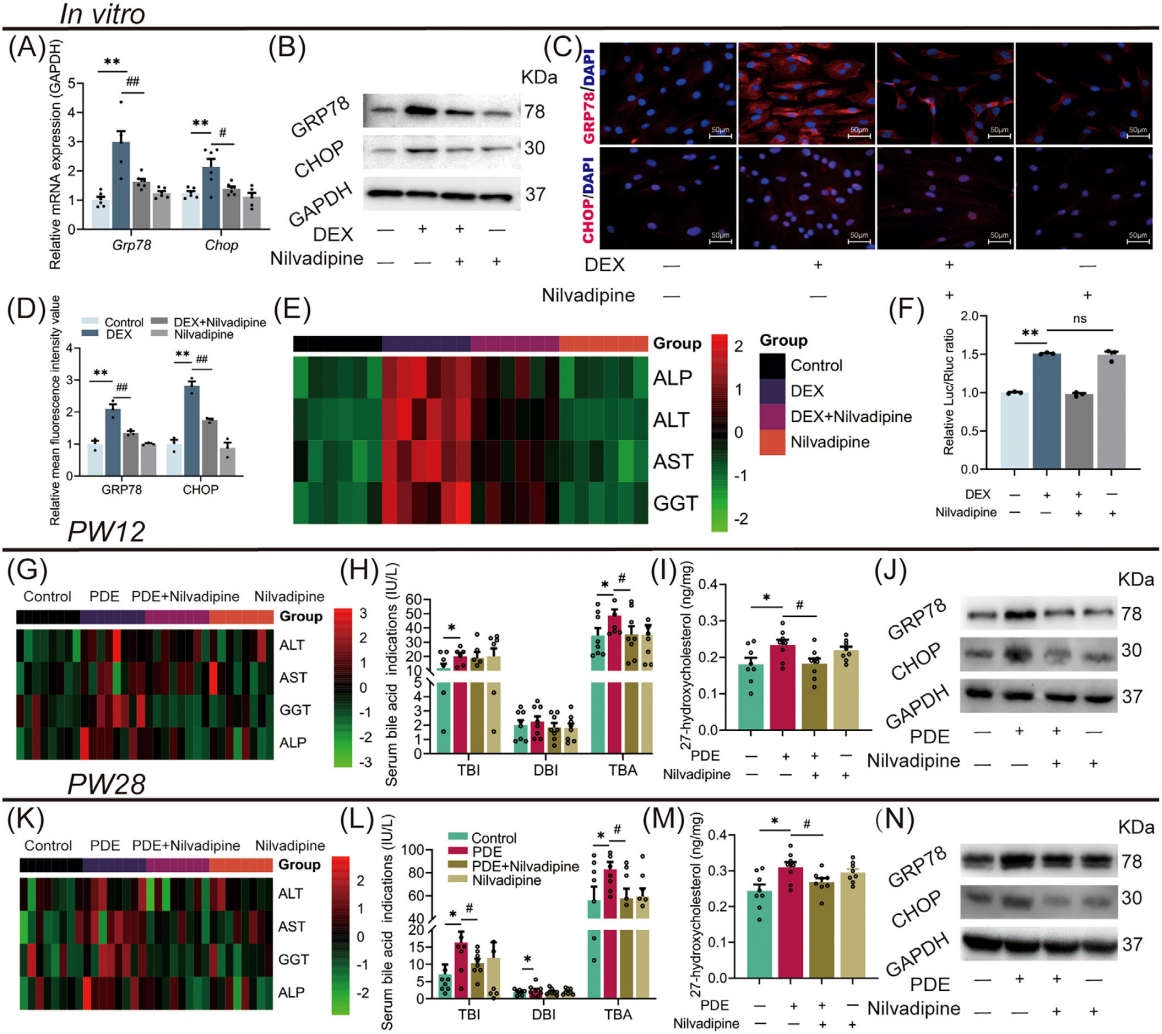
Nilvadipine reversed cholestatic liver injury induced by dexamethasone or PDE in female offspring rats with long‐term efficacy. (A) GRP78 and CHOP mRNA expression. (B) GRP78 and CHOP protein expression by western blot. (C, D) GRP78 and CHOP protein expression by immunofluorescence (400×). (E) Cholestatic liver injury‐related indicators. (F) The relative luciferase activities. (G, K) Liver injury‐related indicators level at PW12 and PW28. (H, L) Cholestasis‐related indicators level at PW12 and PW28. (I, M) Liver 27‐hydroxycholesterol contents at PW12 and PW28. (J, N) GRP78 and CHOP protein expression at PW12 and PW28. A–G was detected in human WJ‐MSCs after being treated with DEX and/or nilvadipine. Data are shown as the mean ± SEM, *n* = 3 for western blot and immunofluorescence, *n* = 10 for detecting the related serum indicators, *n* = 6 for other experiments. ^*^ *p <* 0.05, ^**^ *p <* 0.01 vs. control; ^#^ *p <* 0.05 vs. DEX or PDE group. PDE, prenatal dexamethasone exposure; GRP78, glucose‐regulated protein 78; CHOP, C/EBP homologous protein; GAPDH, glyceraldehyde‐3‐phosphate dehydrogenase; PW, postnatal week; DEX, dexamethasone; ALP, alkaline phosphatase; ALT, alanine aminotransferase; AST, aspartate aminotransferase; GGT, glutamyl transpeptidase; TBI, total bilirubin; DBI, direct bilirubin; TBA, total bile acid; WJ‐MSCs, Wharton's Jelly derived mesenchymal stem cells; DEX, dexamethasone.

Nilvadipine is a potent inhibitor of CYP27A1 activity.^28^ To explore the potential of CYP27A1 as an early intervention target for PDE‐induced CLI, we first observed the effects of nilvadipine on the ERS and CLI induced by dexamethasone at the cellular level. WJ‐MSC‐differentiated hepatoid cells were treated with 2500 nM dexamethasone for 24 h on the 7th day of differentiation and 100 nM nilvadipine for 24 h on the 14th day of differentiation. Compared with the control group, the mRNA/protein expression of GRP78 and CHOP by immunofluorescence (Figure 7A–D), as well as ALP, ALT, AST, and GGT levels in the extracellular fluid (Figure 7E), were increased in the dexamethasone group. However, nilvadipine treatment partially reversed the enhanced ERS and cholestatic hepatocyte injury induced by dexamethasone (Figure 7A–E). Using the luciferase reporter gene system, we found that dexamethasone treatment significantly increased the CYP27A1 transcriptional activity (Figure 7F), but nilvadipine did not alter the effect of dexamethasone. These results suggest that nilvadipine can reduce the enhanced ERS and cholestatic hepatocyte injury induced by dexamethasone via inhibiting CYP27A1 activity rather than regulating its expression.

Meanwhile, we explored the effects of nilvadipine on CLI in female adult offspring rats with PDE by intragastric administration of nilvadipine (1.4 mg/kg·d) for 4 weeks from PW8. Compared with the control group, the PDE group had higher levels of serum ALT, AST, GGT, ALP, TBI, and TBA at PW12 (Figure 7G,H), and the liver 27‐hydroxyl cholesterol (a product of CYP27A1) concentration, and the GRP78 and CHOP protein expression were also increased (Figure 7I,J). However, nilvadipine treatment reversed the above changes induced by PDE (Figure 7G–J). Interestingly, after 16‐week withdrawal of nilvadipine (at PW28), the serum levels of AST, GGT, ALP, TBI, and TBA, the liver concentration of 27‐hydroxyl cholesterol and the GRP78 and CHOP protein expression were still significantly reduced in the female PDE offspring rats compared with those without nilvadipine treatment (Figure 7K–N). In conclusion, CYP27A1 inhibition by nilvadipine for 4 weeks can effectively attenuate CLI in female PDE adult offspring rats, and this treatment has long‐term efficacy.

## 3 DISCUSSION

In clinical practice guidelines, prenatal glucocorticoid therapy significantly improves the survival of preterm infants. However, newborns with dexamethasone therapy before birth often experience significant weight loss, especially with multiple courses.^29^ Our earlier studies in mice showed that dexamethasone exposure at varying stages, doses, and durations affected fetal development. We found that both body length and birth weight were reduced in a manner dependent on the course and dose, with the second trimester showing a more significant impact than the third.^30^ We established a rat model to study developmental toxicity and multiorgan programming effects of dexamethasone exposure during mid‐to‐late gestation, focusing on functional homeostasis changes.^24^, ^25^, ^26^ Abnormal bile acid metabolism is associated with a range of diseases.^32^, ^33^, ^34^, ^35^, ^36^, ^37^ Cholestasis, caused by various factors, is known to induce hepatocyte death and cirrhosis,^11^ with the accumulation of primary unconjugated bile acids in hepatocytes being a significant cause of liver injury.^16^, ^17^, ^18^ Our previous research indicated that PDE could cause maternal intrahepatic cholestasis during pregnancy (ICP) likely related to increased expression of estrogen receptor α (ERα) and CYP7A1 in the maternal liver.^31^ Our untargeted metabolic profiling also showed increased blood bile acid levels in the PDE fetal rats.^14^ In this study, using the above rat model of PDE developmental toxicity, we examined phenotypic changes, intrauterine programming mechanism, early intervention target, and effective drug for CLI in adult offspring rats. We first observed changes in cord blood bile acid levels in neonates treated with dexamethasone before clinical delivery, and similar results were observed in animal experiments. Deoxycholic acid, a toxic bile acid component, can induce ERS,^39^ which leads to liver injury. Our study showed enhanced CDCA and ERS contents in the liver of female PDE offspring rats. In vitro experiments confirmed that CDCA enhanced ERS and promoted cholestasis and liver injury, while the effects were significantly reversed by ERS inhibitor 4‐PBA. These findings suggest that the continuously increased blood CDCA levels before and after birth could induce CLI in the female PDE offspring by enhancing ERS. We further found that PDE‐induced CLI was more pronounced in female offspring, while male offspring showed no significant effects. This sex difference may be due to differential regulation of downstream target genes by dexamethasone interacting with sex hormone receptors.^38^

Intrauterine programming alterations can increase offspring's susceptibility to metabolic syndrome and related diseases.^40^ Bile acid synthesis is a multistep process involving numerous enzymes across different cellular compartments, including the endoplasmic reticulum, mitochondria, cytoplasm, and peroxisomes.^41^ During the intrauterine stage, bile acid synthesis in the liver relies primarily on CYP27A1, the critical enzyme in the acidic pathway, rather than CYP7A1, the key enzyme in the neutral pathway.^21^ The source of CDCA primarily relies on an acidic way mediated by CYP27A1.^42^, ^43^, ^44^ The study identified that elevated levels of primary unconjugated bile acids in the blood and liver of adult female PDE offspring were predominantly due to the prenatal and postnatal overexpression of CYP27A1 in hepatic tissue. Our previous studies also found that PDE inhibited organic anion transporter polypeptide‐related protein 2b1 (Oatp2b1) expression by activating placental GR and inhibited placental farnesoid X receptor (FXR) to reduce breast cancer resistance protein (Bcrp) expression, ultimately to the accumulation of primary unconjugated bile acids in fetal blood.^15^ Additionally, changes in bile acid composition after birth in PDE offspring rats are closely related to decreased gut microbiota involved in intestinal bile acid conversion, elevating the blood levels of “toxic” bile acids.^45^

The molecular mechanism of increased CYP27A1 expression in fetal hepatocytes has not been reported. It is known that glucocorticoids regulate target genes’ expression by promoting GR translocation into the nucleus and binding to their promoter region. GR and target gene DNA interact dynamically, cycling between binding and unbinding states every few sec.^46^ Upon binding to GRE, the GR protein experiences structural alterations, recruits co‐regulatory proteins, and interacts with chromatin‐modifying complexes, thereby modulating the transcription of target genes.^47^, ^48^ We found that PDE increased liver GR protein expression in female PDE fetal rats, along with an increase in the downstream target gene SGK1. In vitro, dexamethasone increased GR protein expression in the nucleus but decreased it in the cytoplasm. Using luciferase reporter gene technology, we confirmed that dexamethasone through active GR binds to GRE sites (between −1094 and −792) in the human CYP27A1 promoter region, significantly increasing CYP27A1 transcriptional activity, and the GR antagonist RU486 reversed this effect. These results suggest that dexamethasone induces CYP27A1 transcription in hepatocytes by activating GR and promoting its binding to the CYP27A1 promoter. This molecular mechanism explains the specificity of GR in inducing high CYP27A1 expression in hepatocytes due to intrauterine dexamethasone exposure.

Epigenetic modifications are pivotal in fetal multiorgan programming, postnatal homeostasis, and the predisposition to diseases of fetal origin.^49^, ^50^, ^51^ In our research, we noted a sustained elevation in H3K14ac marks at the CYP27A1 promoter in liver tissues of female PDE progeny, both prenatally and postnatally. SIRT1, a conserved NAD^+^‐dependent deacetylase, plays a role in hepatic lipid and bile acid metabolism.^52^ We found that the inhibition of SIRT1 expression mediates the dexamethasone‐induced increases in CYP27A1 H3K14ac level and expression. Accumulating evidence indicates that intrauterine programming changes caused by dexamethasone are associated with abnormal miRNA regulation of functional genes.^26^, ^53^ ^−^ ^55^ Through miRNA sequencing and subsequent bioinformatics analysis of fetal liver samples from the PDE cohort, we identified an upregulation of miR‐450b‐3p, which targets the 3′‐UTR region of the SIRT1 mRNA. Consequently, dexamethasone triggers activation of GR in fetal hepatocytes, upregulates miR‐450b‐3p, downregulates SIRT1, and consequently elevates H3K14ac at the CYP27A1 promoter. The cascade of events results in heightened CYP27A1 levels and TBA synthesis, elucidating the epigenetic fetal programming responsible for the enhanced hepatic CYP27A1 in female PDE progeny.

In utero, the maternal–placental–fetal unit constitutes a sophisticated biological and pharmacokinetic entity with intricate interactions, which complicates therapeutic interventions in pregnancy. However, early postnatal intervention may effectively prevent adult diseases. The study found that postpartum exercise can mitigate learning and memory impairments in offspring caused by prenatal morphine exposure.^56^ A fish oil‐rich diet can reverse the adverse reactions caused by a maternal low‐protein diet and reduce blood triglyceride levels and liver steatosis in offspring.^57^ Similarly, administering dimethyl fumarate for 3 weeks postweaning can prevent hypertension in PDE offspring caused by a high‐fat diet.^58^ Our previous finding indicated that abnormal histone acetylation of the ACE promoter mediated PDE‐induced osteogenic differentiation disorder in offspring,^25^ and confirmed that inhibition of ACE expression in the early postnatal period could effectively prevent and treat fetal‐originated osteoporosis.^59^ As previously highlighted, the elevated programming of CYP27A1 expression represents the fetal programming mechanism and a critical toxicological target associated with the development of CLI in the adult female progeny of rats exposed to PDE. Our clinical study also confirmed high expression of CYP27A1 in the PBMC of PDT neonates. Therefore, we speculate that inhibiting CYP27A1 activity may reverse fetal‐originated CLI.

Nilvadipine, a dihydropyridine calcium antagonist, is mainly used to treat angina pectoris, hypertension, cerebral vasospasm, and ischemic heart disease. Studies have shown that nilvadipine can effectively inhibit CYP27A1 activity in mice,^28^ suggesting that nilvadipine is a potential inhibitor of CYP27A1 activity. In this study, nilvadipine can effectively reverse PDE or dexamethasone‐induced 27‐hydroxycholesterol production, TBA and ERS level increases, and adult cholestatic liver injury or hepatocyte injury. Notably, this effect was sustained to PW28 after nilvadipine withdrawal at PW12. These results confirm that CYP27A1 and nilvadipine can serve as early intervention targets and effective treatments for CLI in female PDE offspring, with long‐term efficacy.

While this research comprehensively explored adult disease patterns, fetal programming mechanisms, potential interventions, and therapeutic drugs for cholestasis‐induced liver injury in female PDE offspring through animal and cellular models, clinical studies only established elevated levels of unconjugated bile acids in cord blood of PDT neonates and high CYP27A1 expression in PBMCs. The long‐term effects of PDT on offspring bile acid metabolism were not further assessed, representing a limitation. Future studies will address this gap.

## 4 CONCLUSION

**FIGURE 8:**
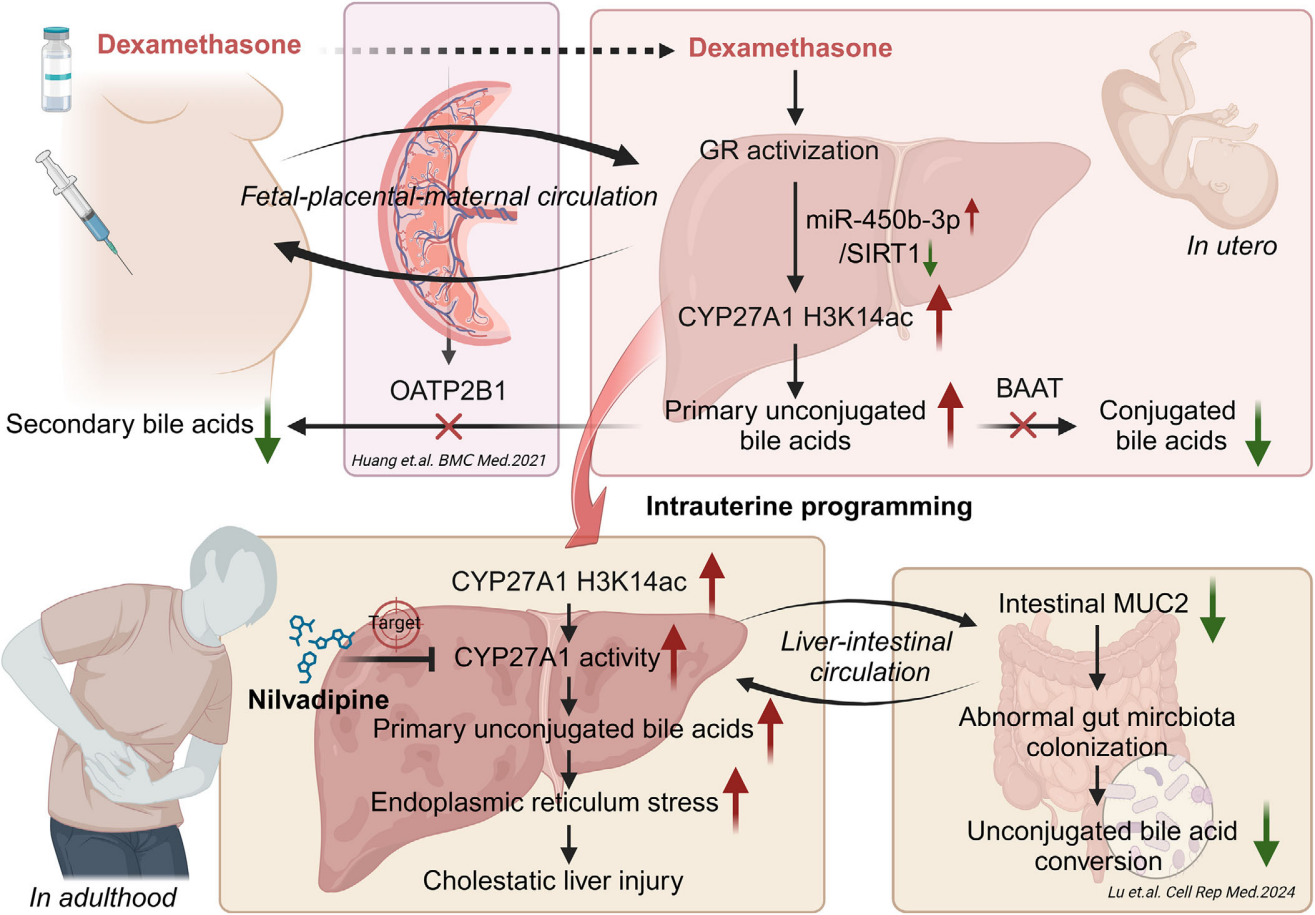
Intrauterine programming mechanisms of cholestatic liver injury induced by prenatal dexamethasone exposure and its drug target. GR, glucocorticoid receptor; SIRT1, Sirtuin 1; CYP27A1, cholesterol 27α‐hydroxylase; Oatp2b1, organic anion transporter polypeptide‐related protein 2b1; BAAT, bile acid‐CoA: amino acid N‐acyltransferase; MUC2x, mucin‐2; BSH, bile salt hydrolase.

Based on clinical PDT neonatal cord blood and fetal blood of PDE rats, this study systematically confirmed for the first time that there is an abnormal metabolic profile of bile acids in fetal blood, mainly manifested as an increase in primary unconjugated bile acid levels. Further, it has been found in the PDE rat model that female adult offspring have a CLI. The mechanism of this occurrence (see Figure 8) is that dexamethasone activates the GR in fetal liver cells. On the one hand, it directly binds to the CYP27A1 promoter to promote its transcriptional expression; on the other hand, it activates the miR‐450b‐3p/SIRT1 pathway to increase the CYP27A1 promoter H3K14ac level and expression. The sustained increases in the H3K14ac level of CYP27A1 promoter and its expression can persist after birth, leading to elevated primary unconjugated cholic acid, enhanced ERS, and CLI in adulthood.

This study has refined the phenomenon of CLI caused by PDE. For the first time, the intrauterine epigenetic mechanism of CLI in the female adult offspring induced by PDE was systematically explained. It was proposed for the first time that CYP27A1 and nilvadipine could be used as early intervention targets and effective medications for cholestasis liver damage in female offspring of PDE, respectively, and they have long‐term efficacy. For the first time, a new intervention method and its optimal intervention time have been identified. These results offer a theoretical foundation and empirical data to inform prudent pharmaceutical administration in pregnancy and to avert conditions that stem from fetal development.

## 5 MATERIALS AND METHODS

### 5.1 Chemicals and reagents

Dexamethasone was sourced from Shuanghe Pharmaceutical, while Nilvadipine was acquired from J&K Scientific. Isoflurane (R510‐22) was supplied by RWD Life Science. Trizol reagent was procured from Invitrogen. The RT‐qPCR kit was provided by TaKaRa Biotechnology, and SYBR Green dye was obtained from Thermo Fisher Scientific via Applied Biosystems. HGF and FGF‐4 were bought from PeproTech. The DNA purification kit was from TIANGEN Biotech. Antibodies targeting H3K9ac, H3K14ac, H3K27ac, SIRT1, CYP7A1, and CHOP were purchased from ABclonal Biotech. The GR antibody was sourced from Cell Signaling Technology and the histone 3 antibody from Service Biotechnology. CYP27A1 and GAPDH antibodies were from Abcam, and GRP78 and 4‐PBA antibodies were from Sigma Aldrich. RIPA lysis buffer and BCA protein assay kit were supplied by Beyotime Biotechnology, and the ECL kit was from Bridgen Biotechnology. All other chemicals and reagents were of analytical grade.

### 5.2 Human study design and blood collection

The project was approved by the Human Ethics Committee of Zhongnan Hospital of Wuhan University, with informed consent from all subjects (no. 2021063). The retrospective analysis (2021–2022) examined medical records from pregnant women and neonates. Inclusion criteria were: (1) Newborns entering the intensive care unit; (2) one course of dexamethasone therapy was given; (3) pregnancies delivered from GW24 to GW42 without administration of dexamethasone. Exclusion criteria: (1) Combined with hypertension, diabetes, heart disease, autoimmune diseases, and other chronic diseases; (2) using other drugs, such as synthetic glucocorticoid prednisone. Participants or guardians provided consent, and the study was ethically approved. EDTA vacutainers were used to collect 3 mL blood samples within 6 h of birth.

### 5.3 Animals and treatment

Pathogen‐free Wistar rats, certified (no. 42000600014526, license SCXK), with female weights of 200 ± 20 g and male weights of 280 ± 20 g, were sourced from the Hubei Medical Scientific Academy's Experimental Center (Wuhan, China). The study protocol was approved by the Committee on the Ethics of Animal Experiments Center of Wuhan University (permit no. 201719). They acclimated to the environment for a week before experiments began. Animals were maintained under standard housing conditions. Mating was verified by the detection of sperm in vaginal smears, designating this day as GD0. PDE groups received subcutaneous injections of 0.2 mg/kg·d dexamethasone from GD9 to GD20; controls received saline. Pregnant rats were sacrificed on GD20. On GD20, pregnant rats were euthanized under 3% isoflurane anesthesia. A separate group of pregnant rats, both PDE and controls, was permitted to give birth naturally at full term. Eight pups from each litter were randomly selected for lactation to ensure balanced nutrition before weaning. At PW4, two female offspring rats were randomly selected from each litter (from 10 litters). These offspring were treated intragastrically with saline or nilvadipine 1.4 mg/kg·d from PW8 to PW12. At PW12 and PW28, respectively, rats were anesthetized and sacrificed to collect blood and liver tissues. The primary detection indicators are as follows:

Biochemical assay to detect CLI‐related indicators in serum: Levels of TBA, ALT, AST, GGT, ALP, DBI, and TBI were quantified using kits by Nanjing Jiancheng Bioengineering Institute, following the protocol provided.
Rhodamine staining to detect cholestasis in tissues: Prepare a stock solution by dissolving 0.1 g of 5‐(p‐dimethylaminobenzylidene) rhodanine in 100 mL absolute ethanol. Prepare the working solution by mixing 20 mL of supernatant from the stock with 30 mL of deionized water. Tissue sections, after deparaffinization and rehydration, are incubated in the rhodanine working solution at 60°C for 1 h. Rinse in deionized water four times, then counterstain with hematoxylin for 10 min, followed by three additional rinses in deionized water.
LC‐MS to detect bile acid metabolites in serum and liver: Thaw serum samples and add 20 to 180 µL of 67% aqueous acetone. Vortex for 30 s and centrifuge at 18,000×*g* for 20 min at 4°C to remove proteins. Analyze using a previously described LC‐MS method for bile acid measurement.^14^ For liver tissue, homogenize 200 mg in 1 mL of physiological saline. Add 10 µL of internal standard to 100 µL of the homogenate, then mix with 1 mL of ice‐cold alkaline acetonitrile. Vortex the mixture and centrifuge at 14,800×*g* for 10 min at 4°C. Reconstitute in 100 µL of MeOH and deionized water (85:15, v/v), centrifuge, and inject 70 µL of supernatant into the LC‐MS system. Analyze using the previously described LC‐MS method for bile acid pattern measurement.

### 5.4 Cell culture and treatment

HepG2 cells were cultured in DMEM with 10% FBS (Gibco), 100 U/mL penicillin, and 100 µg/mL streptomycin at 37°C with 5% CO_2_. Cells were exposed to dexamethasone at 0, 100, 500, and 2500 nM. Additional treatments included 2500 nM dexamethasone, nilvadipine, Nr3c1 siRNA, miRNA‐450b inhibitor, and RU486 for 24 h. Separate treatments involved CDCA (0, 50, 100, 200 µM) or 4‐PBA (1 mM) for 24 h. Cells were then harvested for analysis

Human WJ‐MSCs were cultured in a flask with Iscove's modified Dulbecco's medium (IMDM) containing 1% fetal bovine serum, 1% coumadin antibody, HGF (40 ng/mL), and FGF‐4 (10 ng/mL).^60^ Cells were expanded to the 3rd to 5th generation and differentiated for 21 days. Cells cultured in standard DMEM/F12 medium served as the negative control. After confirming hepatocyte‐like properties, differentiated cells were treated with various concentrations and durations of dexamethasone.

Dil‐Ac‐LDL to detect low‐density lipoprotein absorptivity: Cells were cultured with 15 µg/mL Dil‐Ac‐LDL at 37°C and 5% CO_2_ overnight, then fixed in 4% paraformaldehyde for 10–15 min. After PBS washing and DAPI staining for 15 min, cells were viewed under an inverted microscope.
Periodic acid Schiff's reaction (PAS) staining to detect glycogen synthesis capacity: Cells were fixed with 4% paraformaldehyde for 10–15 min, then washed and treated with periodic acid for 4–6 min. After incubation with Schiff's reagent for 12–16 min and counterstaining with hematoxylin for 90 s, cells were examined under an inverted microscope.
Immunofluorescence to detect liver‐specific protein expression: After fixation in 4% paraformaldehyde for 30 min, cells were permeabilized with 0.2% Triton X‐100 for 15 min and then blocked with 5% goat serum. They were incubated with anti‐albumin or anti‐alpha‐fetoprotein antibodies overnight at 4°C, followed by incubation with Cy3‐conjugated secondary antibodies for 1.5 h. After DAPI staining, cells were visualized under a fluorescence microscope.
Dual‐luciferase assay to verify the targeted interaction between microRNA and mRNA: HepG2 cells were co‐transfected with psiCHECK‐2 and miR‐450b mimics/inhibitors via Lipofectamine 3000. Luciferase activity was assessed 48 h posttransfection using the dual‐luciferase assay (Promega), normalizing to Renilla.

### 5.5 Total RNA extract and RT‐qPCR assay

Steps followed previous lab protocols.^54^ Primers for various genes were listed in Table S2. Gene expression levels were quantified by the comparative 2^−△△Ct^ method, with GAPDH serving as the endogenous control

### 5.6 Western blot assay

Conducted as per lab protocols.^54^ Antibody dilution concentrations were as follows: GR (1:1000), Histone 3 (1:1000), CYP27A1 (1:1000), GAPDH (1:5000), SIRT1 (1:500), CYP7A1 (1:500), GRP78 (1:1000), and CHOP (1:1000).

### 5.7 Immunofluorescence assay

Cells were fixed in 4% paraformaldehyde for 30 min, washed with PBS, permeabilized with 0.2% Triton X‐100 for 15 min, and washed again. Blocking was performed with 5% goat serum for 30 min, followed by incubation with anti‐GRP78 or anti‐CHOP antibodies (1:100 dilution) overnight at 4°C. For liver tissues, samples were fixed in 4% paraformaldehyde, sectioned, and cut into 5 µm slices. Sections were dewaxed, washed, and underwent antigen retrieval before being blocked with 5% serum and incubated with anti‐CYP27A1 antibodies overnight at 4°C. Afterward, Cy3‐labeled secondary antibodies were applied for 1.5 h at room temperature. Finally, sections were washed, DAPI‐stained, and examined under a fluorescence microscope.

### 5.8 ChIP assay

The experimental steps were conducted according to the previous descriptions in our laboratory, and specific details can be found in the referenced literature.^54^ Ultimately, a 2 µL aliquot of DNA was employed in ChIP‐PCR to amplify the CYP27A1 promoter regions, with primer sequences detailed in Table S3.

### 5.9 Sequencing analysis

RNA was isolated using Trizol, and its integrity was verified with both ND‐1000 Nanodrop and Agilent 2200 TapeStation. Ribosomal RNA was removed using the Epicentre Ribo‐Zero kit, and the RNA was subsequently fragmented. Library preparation was conducted with the NEBNext Ultra kit, followed by sequencing on the HiSeq3000. Aligned reads to the rat reference genome were performed with HISAT2, and differential gene expression was determined using DEseq, focusing on genes exhibiting a fold change greater than 2 and an adjusted *p*‐value below 0.05. For small RNA libraries, the NEBNext Multiplex kit was utilized, and sequencing was carried out on the HiSeq 2500 at Ribobio Co. Ltd.

### 5.10 Statistical analysis

Statistical analysis employed SPSS 24.0 (SPSS Science Inc.). Paired Student's *t*‐tests compared two groups, while one‐way ANOVA with post hoc Dunnett's or Bonferroni's *t*‐tests assessed multiple‐group differences. Significance was set at *p *< 0.05, with data expressed as mean ± SEM from at least three independent experiments.

## AUTHOR CONTRIBUTIONS

Wen Hu and Jiayong Zhu prepared the manuscript. Wen Hu, Jiayong Zhu, Qi Zhang, Xiaoqian Lu, Xiaoqian Lu, and Bin Li performed the experiments. Hui Wang and Liaobin Chen planned the project. Hui Wang acquired funding. All authors read and approved the final manuscript.

## CONFLICT OF INTEREST STATEMENT

The authors declare no conflict of interest.

## ETHICS STATEMENT

Adhering to the China Animal Welfare Committee's guidelines, all procedures involving animals were conducted in compliance with the approved protocol by the Wuhan University Animal Experimentation Ethics Committee (permit no. 201719). For the human component, ethical approval was granted by the Zhongnan Hospital of Wuhan University's Human Ethics Committee (no. 2021063). Informed consent was obtained from all subjects for umbilical cord blood collection and subsequent analysis. The study adhered to the Declaration of Helsinki (2013) and ARRIVE Guidelines.

## Supporting information



Supporting information

## Data Availability

The data sets utilized and analyzed in this study are accessible through the principal investigator upon request for legitimate purposes.
