# Supplementary material for: Cholesterol‐27α‐hydroxylase inhibitor nilvadipine can effectively treat cholestatic liver injury in adult offspring induced by prenatal dexamethasone exposure
Source: MedComm (2020). 2025 Mar 4;6(3):e70110. doi: 10.1002/mco2.70110 (PMC11879897; doi:10.1002/mco2.70110)
Supplement: Supplementary file 1 — Supporting information [file MCO2-6-e70110-s001.docx]

**Supplementary data**

**Cholesterol-27α-hydroxylase inhibitor nilvadipine can effectively treat cholestatic liver injury in adult offspring induced by prenatal dexamethasone exposure**

Wen Hu ^1,2,4,#^, Jiayong Zhu ^3,#^, Qi Zhang ^1^, Xiaoqian Lu ^1^, Luting Yu ^1^, Bin Li ^3,4^, Liaobin Chen ^3,4^, Hui Wang ^1,4,*^

^1^ Department of Pharmacology, Wuhan University School of Basic Medical Sciences, Wuhan 430071, China;

^2^ Department of Pharmacy, Zhongnan Hospital of Wuhan University, Wuhan 430071, China;

^3^ Department of Orthopedic Surgery, Zhongnan Hospital of Wuhan University, Wuhan 430071, China;

^4^ Hubei Provincial Key Laboratory of Developmentally Originated Disease, Wuhan 430071, China.

^#^ These authors contributed equally to this study.

^*^Corresponding author: Department of Pharmacology, School of Basic Medical Sciences, Wuhan University, 185 Donghu Road, Wuchang District, Wuhan 430071, China. E-mail address: wanghui19@whu.edu.cn (H. Wang).


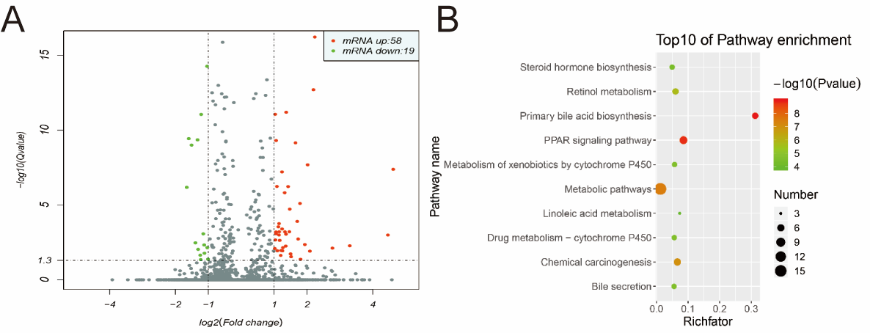


**Figure S1.** **Changes in** **gene expression and KEEG biological pathway enrichment in the liver of female fetal rats with prenatal dexamethasone exposure.** (A) The changed number of gene expressions; **(**B) Analysis of differential gene KEEG biological pathway enrichment. n=1.


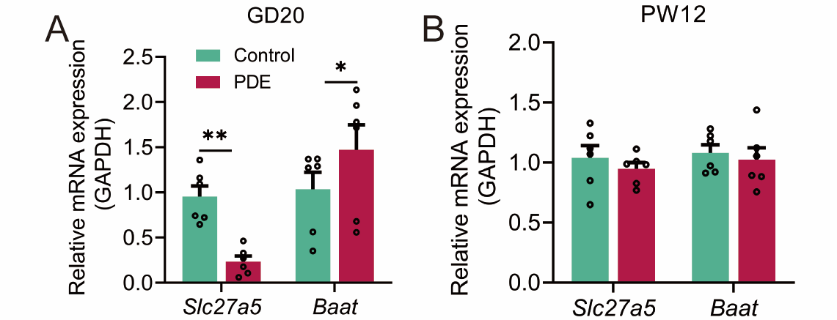


**Figure S2.** **Bile acid transporter expression in the liver of PDE.** (A, B) Bile acid transporter mRNA expression on GD20 and PW12. Data are shown as the mean ± S.E.M., n=6 for RT-qPCR. **P*<0.05 *vs*. control. PDE: prenatal dexamethasone exposure; GD: gestational day; PW: postnatal week; Scl27a5: solute carrier family 27 member 5; Baat: bile acid-CoA: amino acid N-Acyltransferase.


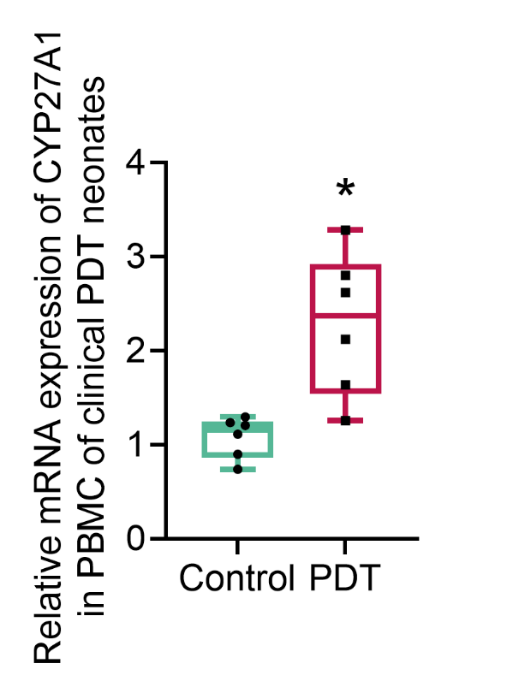


**Figure S3. CYP27A1 expression in PBMC of clinical PDT neonates.** (A) CYP27A1 mRNA expression. Data are shown as the mean ± S.E.M., n=6 for RT-qPCR. **P*<0.05 *vs*. control. CYP27A1: cholesterol 27α-hydroxylase; PBMC: peripheral blood mononuclear cell; PDT: prenatal dexamethasone therapy.


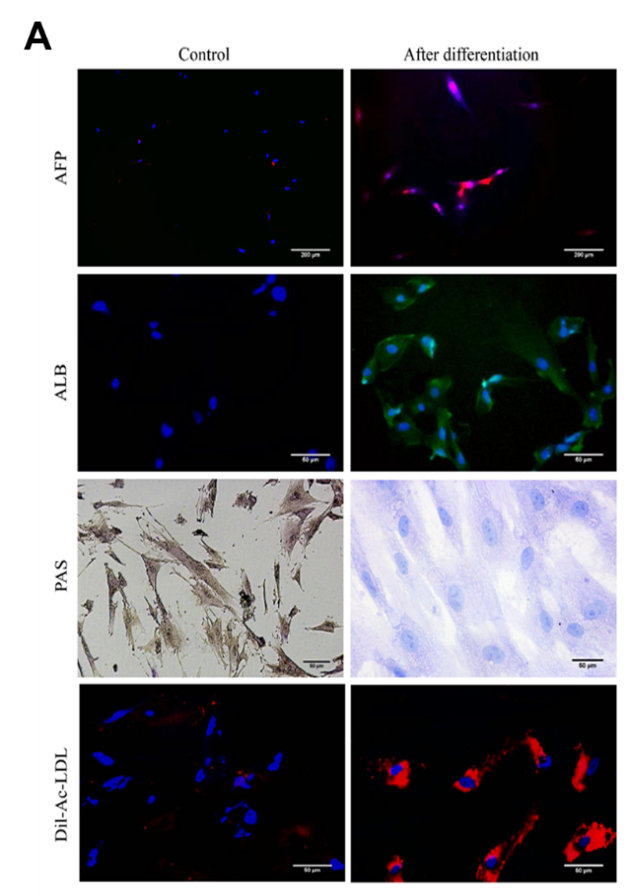


**Figure S4. Human WJ-MSCs are derived from normal newborns to differentiate into hepatocyte-like cells. (**A) The detection of hepatocyte-specific marker expression. n=3. WJ-MSCs: Wharton’s Jelly-derived mesenchymal stem cells; AFP: alpha-fetoprotein; ALB: albumin; PAS: periodic acid-Schiff; Dil-AC-LDL: dil-acetylated low-density lipoprotein.


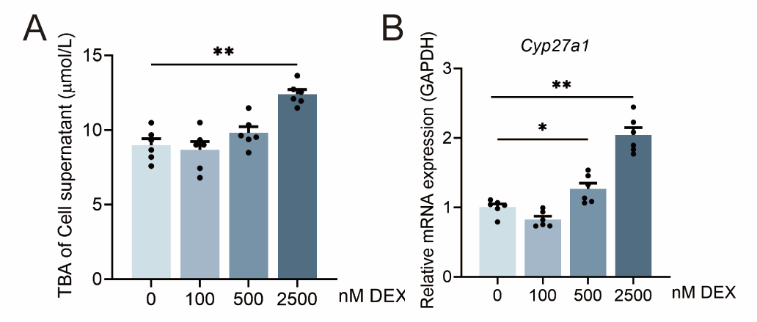


**Figure S5. CYP27A1** **expression and TBA production increase *in HepG2* induced by DEX.** (A) CYP27A1 mRNA expression; (B) TBA level in cell supernatant. Data are shown as the mean ± S.E.M., n=6. ^*^*P*<0.05, ^**^*P*<0.01 *vs*. 0 nM DEX. CYP27A1: cholesterol 27α-hydroxylase; TBA: total bile acid; DEX: dexamethasone; GAPDH: glyceraldehyde-3-phosphate dehydrogenase.


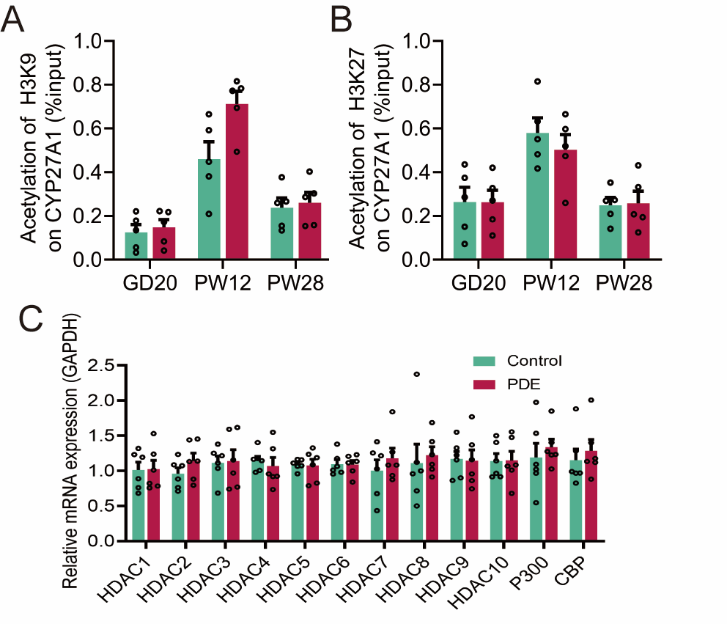


**Figure S6. Changes in** **H3K9ac and H3K27ac levels on CYP27A1 promoter and epigenetic enzymes expression in the liver of female offspring rats with PDE. (**A, B) Enrichment of H3K9ac and H3K27ac in the promoter region of CYP27A1 at GD20, PW12 and PW28; **(**C) The mRNA expression of histone acetylation-related enzymes on GD20. Data are shown as the mean ± S.E.M., n=3 for ChIP assay, n=6 for RT-qPCR. H3K9ac: histone 3 lysine 9 acetylation; H3K27ac: histone 3 lysine 27 acetylation; CYP27A1: cholesterol 27α-hydroxylase; PDE: prenatal dexamethasone exposure; GD: gestational day; PW: postnatal week; HDAC: histone deacetylase; CBP: CREB binding protein; ChIP: chromatin immunoprecipitation; RT-qPCR: real-time quantitative polymerase-chain-reaction.


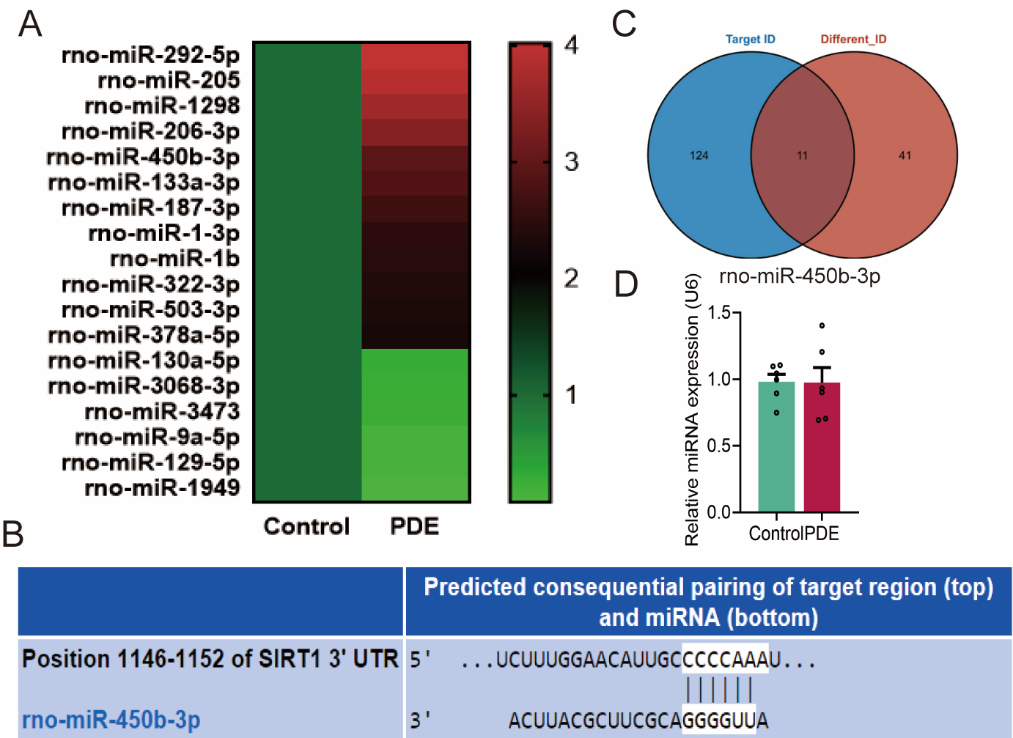


**Figure S7. Changes in the miRNA expression and predictions of the binding site in the liver of female offspring rats with PDE.** (A) Heat map representation of miRNAs differentially expressed at GD20; n=1 for miRNA sequencing experiment; (B) Bioinformatics Analysis; (C) Intersection between differentially expressed miRNA and bindable miRNA (D) The miR-450b-3p expression at PW12. Data are shown as the mean ± S.E.M., n=6 for RT-qPCR. PDE: prenatal dexamethasone exposure; GD: gestational day; PW: postnatal week.


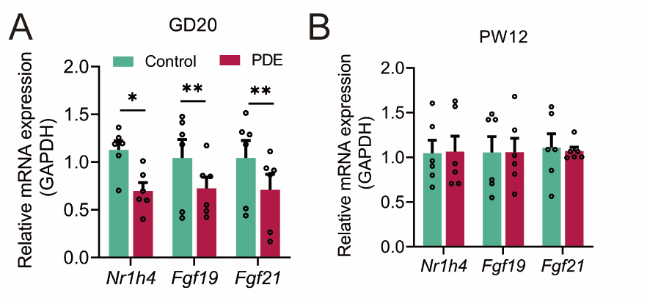


**Figure S8. Genes regulating bile acid synthesis expression in the liver of PDE.** (A, B) Genes regulating bile acid synthesis mRNA expression on GD20 and PW12. A Data are shown as the mean ± S.E.M., n=6 for RT-qPCR. **P*<0.05 *vs.* control. PDE: prenatal dexamethasone exposure; GD: gestational day; PW: postnatal week; Nr1h4: nuclear receptor subfamily 1 group H member 4; Fgf19: fibroblast growth factor 19; Fgf21: fibroblast growth factor 21.


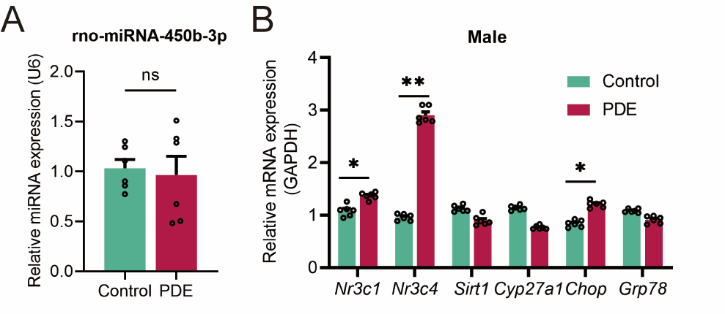


**Figure S9.** **GR/miRNA-450b-3p/Sirt1/CYP27A1 pathway expression in the liver of PDE.** (A) The miR-450b-3p expression at GD20 of PDE male offspring; (B) GR/miRNA-450b-3p/Sirt1/CYP27A1 pathway mRNA expression at GD20 of PDE male offspring. Data are shown as the mean ± S.E.M., n=6 for RT-qPCR. **P*<0.05 *vs*. control. PDE: prenatal dexamethasone exposure.


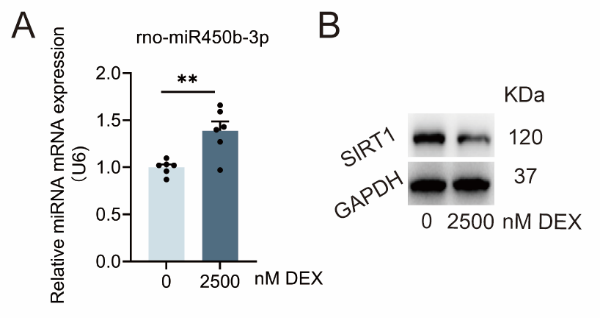


**Figure S10. miR-450b-3p and SIRT1** **expression changes *in HepG2* induced by DEX.** (A) miR-450b-3p expression after DEX treatment; (B) SIRT1 protein expression after DEX treatment. Data are shown as the mean ± S.E.M., n=6 for RT-qPCR.SIRT1: Sirtuin 1; DEX: dexamethasone; GAPDH: glyceraldehyde-3-phosphate dehydrogenase.

**Table S1.** **Clinical data of neonates and their mothers in control and prenatal dexamethasone therapy (PDT) groups.**

|  | Male | | | Female | | |
| --- | --- | --- | --- | --- | --- | --- |
|  | CON | PDT | Significance | CON | PDT | Significance |
|  | (N=25) | (N=20) |  | (N=21) | (N=14) |  |
| Gestation at Delivery (weeks+days) | 33^+2^ (31^+2^-34^+6^) | 33^+4^ (32^+2^-34^+4^) | N.S. | 33^+1^ (31^+6^-34^+3^) | 33^+2^ (32^+1^-34^+3^) | N.S. |
| Age (y) | 32±5.4 | 34±6.1 | N.S. | 32±6.1 | 34±5.3 | N.S. |
| Height (cm) | 158±5.5 | 161±4.1 | N.S. | 162±5.8 | 162±3.8 | N.S. |
| Weight (kg） | 71±9.4 | 73±10.8 | N.S. | 73±9.6 | 69±9.7 | N.S. |
| History of liver disease | 2 | 1 | N.S. | 1 | 1 | N.S. |
| TBA(µmol/L) | 2.28±0.81 | 2.47±1 | N.S. | 3.44±1.96 | 3.64±2.56 | N.S. |
| ALT(U/L) | 9.21±3.43 | 14.39±8.98 | N.S. | 9.29±2.99 | 12±2.49 | *P＜*0.05 |
| AST(U/L) | 16.75±4.12 | 23.3±9.11 | *P＜*0.05 | 15±2.76 | 18±3.93 | *P＜*0.05 |
| ALP(U/L) | 206.92±86.17 | 143.13±34.9 | N.S. | 199±80.18 | 158.5±64.02 | N.S. |
| GGT(U/L) | 11.33±4.41 | 15.6±13.26 | N.S. | 11.29±5.95 | 10.25±3.89 | N.S. |
| TBIL(µmol/L) | 8.9±3.81 | 9.34±10.26 | N.S. | 8.83±3.89 | 8.25±2.55 | N.S. |
| UBIL(µmol/L) | 1.54±0.73 | 1.03±0.43 | N.S. | 1.32±0.74 | 1.5±0.62 | N.S. |
| IBIL(µmol/L) | 7.35±3.85 | 5.97±1.89 | N.S. | 7.51±3.7 | 6.75±2.51 | N.S. |
| BUN(mmol/L) | 4.2±1.23 | 4.29±2.04 | N.S. | 4.34±0.9 | 3.94±1.04 | N.S. |
| CREA(µmol/L) | 45.37±8.24 | 53.86±15.87 | N.S. | 47.98±6.23 | 53.6±11.49 | N.S. |
| GLU(mmol/L) | 4.91±1.14 | 4.3±0.88 | N.S. | 4.8±1.22 | 4.4±1.73 | N.S. |
| Birth weight (g) | 2358 ± 97 | 2267 ± 92 | N.S. | 2458 ± 89 | 2379 ± 77 | N.S. |
| Apgar scores (1min) ^a^ | 9 (7-9) | 8 (7-9) | N.S. | 8 (6-9) | 8 (7-9) | N.S. |
| Apgar scores (5min) | 10 (8-10) | 10 (8-10) | N.S. | 10 (8-10) | 10 (8-10) | N.S. |

Data are shown as mean ± S.E.M.

The statistical analysis of newborn body weight, body length, head circumference and Apgar score in the control group and PDT group showed that compared with neonates in the control group, the body weight and body length of male neonates in the PDT group were significantly decreased (Table S1). Still, there were no significant changes in head circumference and Apgar score (Table S1). The body weight of female neonates in the PDT group was significantly decreased (Table S1), but body length, head circumference and Apgar score were not significantly changed.

**Table S2. Oligonucleotide primers were used for quantitative real-time PCR (rat).**

| Genes | Forward primer | Reverse primer | Annealing |
| --- | --- | --- | --- |
| GAPDH (rat) | GCCTCCAAGGAGTAAGAAAC | GTCTGGGATGGAATTGTGAG | 60℃, 30 s |
| CYP27A1 (rat) | ACTGCACCAGTTACAGGTGCTTTACA | CCATGTCGTTCCGTACTGGGTACT | 60℃, 30 s |
| CYP7A1 (rat) | CCATAAGGTGTTGTGCCACGGAAA | GCCCAAATGCCTTCGCAGAAG | 60℃, 30 s |
| MRP2 (rat) | AACCGGGAAGGTCAAGTTCTCCAT | TTGTCAGAGTCACTGGTCCAAGCA | 60℃, 30 s |
| BSEP (rat) | ATCTGTTAATCCTGGGCAGACGCT | TGGGAGACAATCCCGATGTTGGAA | 60℃, 30 s |
| NTCP (rat) | AGGATGGAGGTGCACAACGTATCA | AGCCCAGTGAGAGCATGATAAGCA | 60℃, 30 s |
| SIRT1 (rat) | CAGGTCAAGGGATGGTATTTATG | CAGCGTGTCTATGTTCTGGGTAT | 60℃, 30 s |
| GR (rat) | CACCCATGACCCTGTCAGTC | AAAGCCTCCCTCTGCTAACC | 63℃, 30 s |
| GRP78 (rat) | TCAGCCCACCGTAACAATCAAG | TCCAGTCAGATCAAATGTACCCAGA | 60℃, 30 s |
| CHOP (rat) | TGGAAGCCTGGTATGAGGATCTG | GAGGTGCTTGTGACCTCTGCTG | 60℃, 30 s |
| HDAC1 (rat) | GTGGCCCTGGA CACAGAGAT | GCTTGAAATCTGGTCCAAAGT | 63℃, 30 s |
| HDAC2 (rat) | CAACCTAACTGTCAAAGGTCAC | TGAAGTCTGGTCCAAAATACTCG | 62°C, 30 s |
| HDAC3 (rat) | AGGTGGTGGACTTCTATCAG | CACCAGGAGAGGGATATTGA | 58°C, 30 s |
| HDAC4 (rat) | CTACATCAGAGACCCAATGC | GTGACTGTCTCAGCTTCTTC | 60°C, 30 s |
| HDAC5 (rat) | CCGTGCTCTACATCTCTTTG | GCTGTCAGGTATTCCACATC | 58°C, 30 s |
| HDAC6 (rat) | AGGGAACTACACTGGATCTG | GGCATTTGAGGATGGAGAAG | 60°C, 30 s |
| HDAC7 (rat) | TACAGAACTCTTGAGCCCTT | CAGGGATTTCTTGGGTTTGT | 60°C, 30 s |
| HDAC8 (rat) | GGCAAGTGTCTGAAGTATGT | TGGGATCTCAGAGGATAGTG | 60°C, 30 s |
| HDAC9 (rat) | CCCAGCATCCTGTACATTTC | GCTTCAAGGTACTCAACATCTC | 60°C, 30 s |
| HDAC10 (rat) | CTTCACAAGTCCCAGTTTCA | ATTCTCCTCTGACCTCTATGG | 60°C, 30 s |
| HDAC11 (rat) | CTTACTTCCTCCCTTCAGTCT | CCTTCTTCAGACCTCCAAATC | 60°C, 30 s |
| P300 (rat) | CAAATGCAGGCATGGGCAAT | TCCTGGTTGTCCTCCCATCT | 60°C, 30 s |
| CBP | GACCAAGATGGGGATGACTG | CCACTGATGTTTGCAACTGG | 60°C, 30 s |
| AKR1C1 (rat) | GCTGGAGAAGATCCTGAATAAG | CCAGAGCACTATAAGCAACC | 60°C, 30 s |
| AKR1D1 (rat) | GGTTGGTTGTCATCCCTAAA | GATCACTCCACATGAGCATC | 60°C, 30 s |
| CH25H (rat) | CCACTCAGGTTACGACTTTC | TTTGTCCCAGTGTGTGAAG | 60°C, 30 s |
| CYP46A (rat) | GGATTGGGCTAAGAAGTATGG | CTCCGAACACAGTCTGAATC | 60°C, 30 s |
| CYP39A1 (rat) | ATGAAAGGGAAGATGGGAAC | GAGTGTGGCTGGATAAAGTAG | 60°C, 30 s |
| HSD3B7 (rat) | TGTGGACTTCGGCTGATA | TGACAGTAAAGGTGGTGTTG | 60°C, 30 s |
| GAPDH (human) | TGGATGATGCCTTTGCTCGT | GGGAACTTGCAGTACTCCCC | 60℃, 30 s |
| CYP27A1 (human) | GATTGCAGAGCTGGAGATG | GCAGGCCCACTTTCTTATT | 60℃, 30 s |
| SIRT1 (human) | TAGCCTTGTCAGATAAGGAAGGA | ACAGCTTCACAGTCAACTTTGT | 60℃, 30 s |
| GRP78 (human) | GAA CAT CCT GGT GTT TGA CC | CCC AGA TGA GTA TCT CCATT | 58℃, 30 s |
| CHOP (human) | CTC TGG CTT GGC TGA CTG A | GCT CTG GGA GGT GCT TGT | 58℃, 30 s |

GAPDH: glyceraldehyde 3-phosphate dehydrogenase; CYP27A1: cholesterol 27-hydroxylase; CYP7A1: cholesterol 7α-hydroxylase; MRP2: multidrug resistance-associated proteins 2; BSEP: bile salt export pump; NTCP: Na^+^-dependent taurocholate transporter; SIRT1: Sirtuin1; GR: glucocorticoid receptor; GRP78: glucose-regulatory protein 78; CHOP: C/EBP homologous protein; HDAC: histone deacetylase; CBP: CREB-binding protein; AKR1C1: aldo-keto reductase family 1, member C1; AKR1D1: aldo-keto reductase family 1, member D1; CH25H: cholesterol 25-hydroxylase; CYP46A1: cytochrome P450 family 46 subfamily A member 1; CYP39A1: cytochrome P450 family 39 subfamilies A member 1; HSD3B7: hydroxy-delta-5-steroid dehydrogenase, 3 beta- and steroid delta-isomerase 7.

**Table S3. Human and rat cholesterol 27-hydroxylase (CYP27A1) primers were used for ChIP-PCR.**

| Genes | Forward primer (5’-3’) | Reverse primer (5’-3’) | Annealing |
| --- | --- | --- | --- |
| CYP27A1 (human) | GGGAGTGGGGAGGAGAGAAT | TCTCCTTTCCCGGGACTTCA | 60℃, 30 s |
| CYP27A1 (rat) | GAGCCGCATCTATAGCCCTG | TGGTTGGTTTGAAGCTGGGT | 60℃, 30 s |
